# Complementarity of International Instruments in the Field of Biosecurity

**DOI:** 10.3389/fpubh.2022.894389

**Published:** 2022-05-27

**Authors:** Iris M. Vennis, Maja Boskovic, Diederik A. Bleijs, Saskia A. Rutjes

**Affiliations:** ^1^Centre for Infectious Disease Control, Laboratory for Zoonoses and Environmental Microbiology, National Institute for Public Health and the Environment, Bilthoven, Netherlands; ^2^Biosecurity Office, National Institute for Public Health and the Environment, Bilthoven, Netherlands; ^3^Enconet Consulting Ges.m.b.H., Vienna, Austria

**Keywords:** global health security, biosecurity, legal instruments, health policy, infectious disease, IHR, BTWC, UNSCR1540

## Abstract

The COVID-19 pandemic has demonstrated the devastating impact of infectious disease outbreaks and the threat of emerging and re-emerging dangerous pathogens, independent of their origin. Natural, accidental, and deliberate disease outbreaks all need systems in place for an effective public health response. The best known international instrument in the field of public health is the WHO International Health Regulations (2005). Although the International Health Regulations are mainly focused on natural disease outbreaks, the actions to take to comply with them also contribute to biosecurity and non-proliferation. This paper examines in case of full implementation of the International Health Regulations, what other actions states should take to comply with international biosecurity instruments, including the Biological and Toxin Weapons Convention and United Nations Security Council Resolution 1540, to effectively prevent and defend against intentional biological threats. An overview of international instruments from different disciplines regarding biosecurity is presented. Furthermore, this paper clarifies the similarities between the international biosecurity instruments and addresses the additional requirements that instruments stipulate. From a detailed comparison between the instruments it can be concluded that, to adhere to all legally-binding international biosecurity instruments, specific non-proliferation and export control measures are necessary in addition to full implementation of the International Health Regulations. Additionally, an overview of non-legally binding instruments in the field of biosecurity is presented and practical implementation examples are highlighted. Compliance with legally binding instruments can be improved by precise guidance provided by non-legally binding instruments that are clear and attuned to the situation on the ground. To improve understanding of the existing international instruments, this paper aims to provide an overview of the international legal biosecurity framework to biosecurity experts, policymakers, civil servants, and practitioners. It offers possible practical applications for the politico-legal context and accommodates the enhancement of full employment of biosecurity resources for an improved multidisciplinary capacity to prevent, detect, and respond to infectious disease outbreaks.

## Introduction

The coronavirus SARS-CoV-2 causing the COVID-19 pandemic has stimulated global attention to the impacts of infectious disease and global health security threats. The virus emerged in Wuhan China in late 2019 presenting a local public health challenge (1), but quickly transformed into a global health emergency as the World Health Organization (WHO) declared the outbreak a Public Health Emergency of International Concern (PHEIC) (2), the highest level of alarm, in late January 2020 and a global pandemic in March of the same year (3). This rapid transition from a local outbreak to pandemic status raises questions about the systems in place to prevent and control infectious disease outbreaks. The COVID-19 pandemic is not the only PHEIC of this century. Since 2009, there have been five more PHEIC declarations: the 2009 H1N1 (swine flu) pandemic, the 2014 polio declaration, the 2014 outbreak of Ebola in Western Africa, the 2015–2016 Zika virus epidemic, and the 2018–2020 Kivu Ebola epidemic (4).

In October 2019, the Global Health Security Index analysis found no country to be fully prepared for epidemics or pandemics (5). The report states many countries do not show evidence of the health security capacities and capabilities that are needed to prevent, detect, and respond to significant infectious disease outbreaks. The COVID-19 pandemic demonstrated that the world collectively did not have sufficient capacity to prevent and control major infectious disease outbreaks (6). The SARS-CoV-2 outbreak causes serious reevaluations about the dangers of natural, accidental and deliberate disease outbreaks (7). The 2021 Global Health Security analysis shows all countries remain dangerously unprepared for future epidemic and pandemic threats, including threats potentially more devastating than COVID-19 (8). As with previous PHEICs, the COVID-19 pandemic again revealed that global collaboration and information sharing is critical, as infectious diseases do not stop at country borders (9). This emphasizes the necessity of global health diplomacy, which is at the intersection of public health and foreign affairs (10). A multidisciplinary and global approach is crucial to efficiently prevent and control pandemics. Sustained interaction between biosafety and biosecurity regimes strengthens the international systems for countering disease threats, regardless of their origins (11, 12). This underlines the need for improved implementation of cross-sectional international regulations and systems concerning global health security. Experts have warned for an urgent need to strengthen international arrangements intended to protect the world against chemical, biological, radiological, and nuclear threats (13).

The COVID-19 pandemic has increased attention toward the WHO's International Health Regulations 2005 (IHR) (14). One of the requirements of the IHR is for states to build effective disease surveillance capacities and to notify WHO immediately if an event is considered a public health crisis with the potential of international spread (14). The IHR focuses on infectious disease outbreaks with a natural origin and covers some aspects of accidental and deliberate releases. However, independent of the origin of a disease outbreak, an effective public health response is necessary to control it (15). Health surveillance (prevention of natural outbreaks), biosafety (prevention of accidental release), and biosecurity (prevention of misuse of biological agents and knowledge, with a focus on non-state actors) strive toward reducing public health risks and the means to reach this goal are largely similar. Obligations stemming from the IHR therefore also contribute to biosecurity and non-proliferation (preventing and controlling the spread of weapons of mass destruction, with a focus on state-actors) and, *vice versa*, measures from international non-proliferation instruments contribute to a reduced risk of natural outbreaks. The web of prevention for biosafety and biosecurity provides insight in this complementary relationship (11). The Biological and Toxin Weapons Convention (BTWC) and United Nations Security Council Resolution 1540 (UNSCR1540) are international legally binding non-proliferation instruments in place to reduce dangers of deliberate disease outbreaks in humans, animals, and plants (16). The BTWC also contributes to global disease surveillance as it requests international exchange of equipment, materials, and information to combat outbreaks of infectious diseases (17). UNSCR1540 emphasizes safe and secure handling, use, transport, and storage of pathogenic material, thereby contributing to biosafety and biosecurity (18). As the means to reduce the risk of natural, accidental, and deliberate disease outbreaks are similar, requirements stemming from the various international instruments show overlap. This paper focuses on the obligation stemming from IHR, BTWC, and UNSCR1540 regarding biosecurity. IHR is well-known by most countries and to date 174 of the 196 states parties (89%) submitted an annual IHR self-assessment report over 2020 (19). Therefore, this study examines what else should be in place to comply with international legally binding instruments in the field of biosecurity assuming a fully implemented IHR. Overlap in the requirements of IHR, BTWC, and UNSCR1540 with regard to biosafety and biosecurity has been previously described by Bakanidze et al. (16) and UPMC Center for Health Security published a synopsis of biological safety and security arrangements providing an overview of key international treaties, agreements, instruments, and guidelines (20). This paper builds on previously published work by providing a detailed and updated comparison of the specific requirements stated in each instrument. An up to date overview of legally binding and non-legally binding instruments in the field of biosecurity is given and overlap in the requirements of the legally binding instruments IHR, BTWC, and UNSCR1540 regarding biosecurity is discussed in detail. Requirements stemming from each of these instruments are compared on the level of exact wording of the convention, regulation or resolution in order to know more precisely what additional requirements BTWC and UNSCR1540 require regarding biosecurity when IHR is fully implemented. Furthermore, practical implementation examples are highlighted. This paper aims to facilitate identification of overlapping and complementary issues in international biosecurity instruments and improve understanding of policymakers, civil servants, biosecurity experts, and practitioners regarding these instruments. This accommodates the enhancement of full employment of national resources to comply with international requirements, ultimately leading to an improved capacity to prevent, detect, and respond to infectious disease outbreaks, independent of their origin.

## International Legally Binding Instruments

### IHR

The International Health Regulations represent “An agreed code of conduct adopted by the World Health Assembly in May 2005 to protect against the spread of serious risks to public health and, the unnecessary or excessive use of restrictions in traffic or trade” (21). These regulations are legally binding instruments for all WHO's Members, unless rejection or reservations are formally stated. The IHR aims to ensure a rapid gathering of information, a common understanding of what may constitute a public health emergency of international concern and the availability of international assistance to countries (14). A key element of the IHR is the requirement to notify the WHO if an event is considered to constitute a public health risk to other states through the international spread of disease and potentially require a coordinated international response. Furthermore, the IHR states WHO's responsibility to recommend measures for implementation for each specific emergency (22). The IHR also sets requirements for national core capacities, and member states are obliged to develop capacities to detect, assess, report, notify and respond promptly and effectively to public health events. The implementation of the IHR is a long-term process that calls for states to develop and strengthen specific national public health capacities, identify priority areas for action, develop national IHR implementation plans, and maintain these capacities and continue to build and strengthen as needed over time.

A Monitoring and Evaluation Framework was developed to provide states with a roadmap for assessing their current public health capacities, thus, enabling them to identify areas where improvement is needed, as well as adequate measures that are required for achieving a satisfactory level of capacities for the management of public health risks and emergencies. Although the framework is composed of four processes, the Joint External Evaluation (JEE) is the most apparent. The JEE is a voluntary and comprehensive process aimed at evaluating country's public health capacity across 19 technical areas in a collaborative effort between the country's own experts and the external evaluation team (23). The JEE creates a baseline assessment, enabling countries to have a greater understanding of their gaps and weaknesses in health security as well as to prioritize their efforts for closing those gaps. Although JEE has limitations in accuracy and consistency across the JEE process, JEE provides an informative, and practical assessment of IHR obligations.

### BTWC

The Biological and Toxin Weapons Convention (formally known as the Convention on the Prohibition of the Development, Production, and Stockpiling of Bacteriological (Biological) and Toxin Weapons and on their Destruction) was opened for signature on 10 April 1972 and entered into force 3 years later (17). The Convention prohibits to its contracting parties the development, production, stockpiling, or other ways of acquiring or retaining biological and toxin weapons or their means of delivery and requires that states prevent and prohibit the same activities within their territory, under their jurisdiction or anywhere else under their control. However, it does not prohibit peaceful microbiological activities, including the international exchange of microbial or other biological agents, or toxins and equipment for the processing, use or production of biological agents and toxins for prophylactic, protective, or other peaceful purposes.

The convention has been supplemented by the contracting parties through approval of a series of additional agreements and understandings at the Review Conferences. They either interpret, define or elaborate the meaning or scope of a provision of the Convention, or provide instructions, guidelines or recommendations on how a provision should be implemented. One of the interpretations and instructions these agreements have introduced in relation to Articles I–IV that require specific national “transposing” measures, are confidence building measures (CBM). At the Second Review Conference the states parties agreed to implement a number of CBMs in order to prevent or reduce the occurrence of ambiguities, doubts and suspicions, with the aim to improve international co-operation and transparency in the field of peaceful biological activities. Although these measures are not derived directly from the text of the Convention itself, participation in the CBMs is a requirement for all states parties to the Convention.

Another important additional agreement was the establishment of an Implementation Support Unit (ISU) with the mandate to assist the states parties in implementation of the Convention. The ISU provides administrative support and assistance, national implementation support and assistance, administers the database for assistance requests, and offers and facilitates associated exchanges of information. ISU also provides support and assistance for CBMs, and support and assistance for obtaining universality of the Convention. Furthermore, it supports states parties' efforts to implement the decisions and recommendations of the Review Conferences.

### UNSCR1540

In 2004 the UN Security Council adopted under Chapter VII of the UN Charter Resolution 1540. By this resolution the UN Security Council obliged all states to refrain from providing any form of support to non-state actors that attempt to develop, acquire, manufacture, possess, transport, transfer or use nuclear, chemical or biological weapons and their means of delivery, and to adopt and enforce appropriate effective laws which prohibit any non-state actor to attempt, engage, participate, assist, or finance the foregoing activities. Under resolution 1540 countries should take and enforce effective measures to establish domestic controls to prevent the proliferation of nuclear, chemical, or biological weapons and their means of delivery. This includes establishing appropriate controls over related materials by means of (1) developing and maintaining appropriate effective measures to account for and secure such items in production, use, storage or transport; (2) developing and maintaining appropriate effective physical protection measures; (3) developing and maintaining appropriate effective border controls and law enforcement efforts to detect, deter, prevent and combat, including through international cooperation when necessary, the illicit trafficking; (4) establishing, developing, reviewing and maintaining appropriate effective national export and trans-shipment controls over such items, including appropriate laws and regulations to control export, transit, trans-shipment and re-export and controls on providing funds and services.

Through resolution 1540, the UN Security Council called upon all states to present to the 1540 Committee a national report on steps they have taken or intend to take to implement this resolution. In the following years the Security Council adopted several new resolutions under Chapter VII whose aims were among others to restate the obligations stemming from Resolution 1540, urge its full implementation, call for further voluntary measures to be implemented (e.g., development of national action plans) and broaden the mandate of the 1540 Committee. These are Resolutions 1673 (2006), 1810 (2008), 1977 (2011), 2325 (2016), 2572 (2021), and 2622 (2022) (24–29).

## Requirements for Compliance With Legally Binding International Instruments

In order to create insight in the actions to take to comply with the internationally-mandated requirements, the obligations stemming from IHR, represented by the JEE, UNSCR1540, and BTWC are compared in detail. In order to make a comparison, obligations stemming from the legally binding instruments were grouped, taking into account different methods.

**Figure 1:**
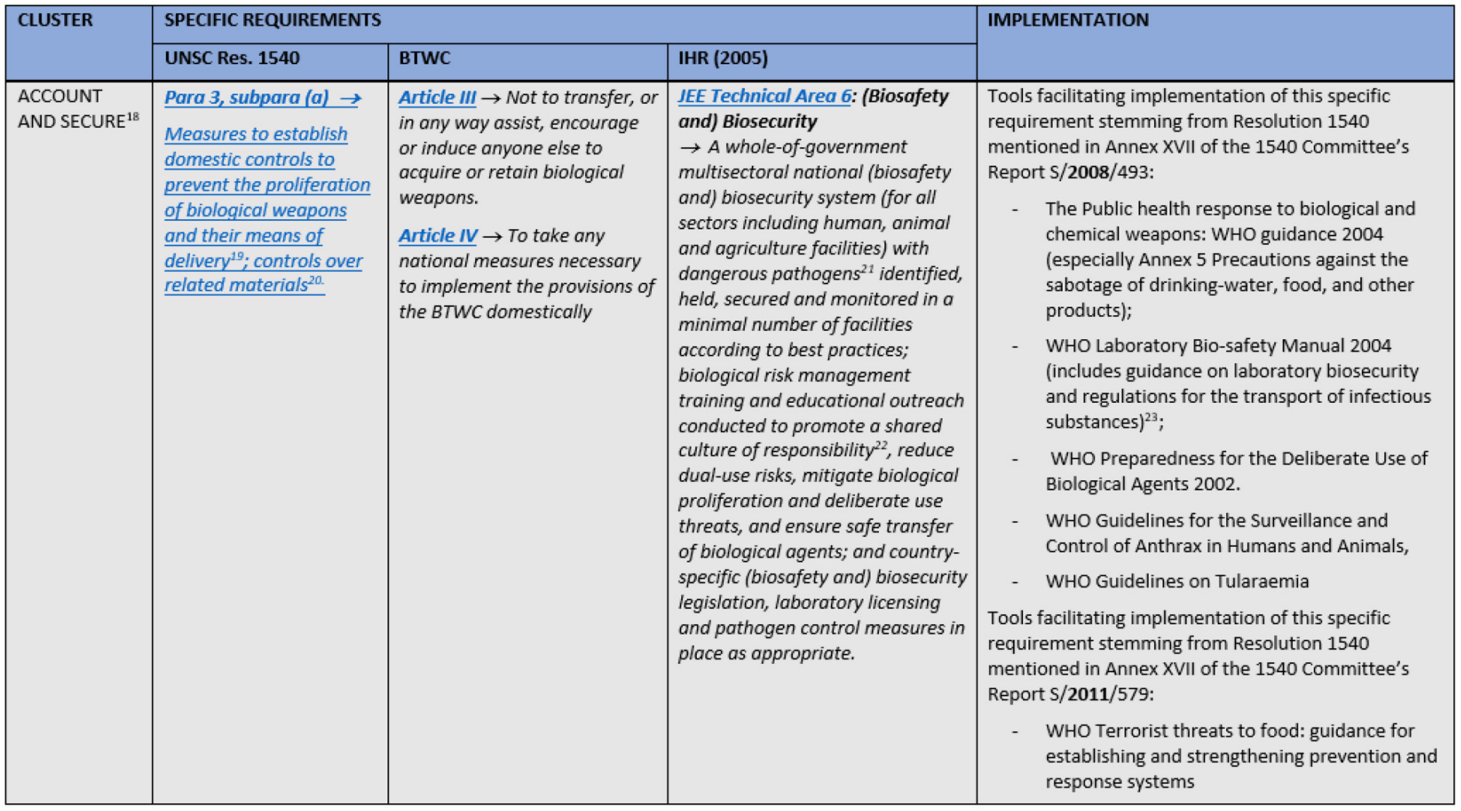
A short section of the matrices with obligations stemming from UNSCR1540, BTWC, and IHR and an implementation example.

A combination of the JEE's groups and similar tasks from the other two instruments were used to cluster the obligations into five fields of action: prevention; prohibition and penalties; detection; response; and international cooperation. The JEE's structure was selected as the basis for the comparison tables' (matrices) skeleton for several reasons. First of all, Resolution 1540 and BTWC are not divided in thematic sections. Therefore, one could either choose the only existing thematic division, or design a new one. Instead of inventing the wheel, we choose to use the JEE's structure which is at the same time the most natural grouping of measures for acting upon an emergency—one needs to prepare for it, to try to detect it and if it happens to respond to it. In addition to all this, it should be noted that the Joint External Evaluation Tool for IHR has the most extensive number of requirements (23) with detailed description of the ways and ranges within which these requirements may be implemented. This naturally makes it easier to “subsume” BTWC's and Resolution's requirements under its thematic groups or to align them with specific requirements from the JEE within one table. What was added to the JEE's structure as a direct consequence of the Resolution's and Convention's substance are the clusters on prohibitions and penalties, and on international cooperation; former containing exclusively requirements from the Resolution and Convention, while the latter has also some from the JEE which are originally within JEE's detection and response thematic groups. It was decided to position cluster on prohibitions and penalties immediately after prevention, as proscribing certain activities and setting appropriate civil and criminal penalties for them acts as a deterrent, i.e., can be considered as a preventive measure. Cluster international cooperation is the last one in the matrix as this is considered to be the “add on” to the national efforts. Hence, to compare the three legally binding instruments on these fields of action (clusters), a matrix was created for each field (Supplementary Tables 1–5). Each matrix is split into columns that include specific requirements stemming from UNSCR1540, BTWC and JEE. A short section of the matrices is displayed in Figure 1.

The matrices also include references to or extracts from non-legally binding international instruments, guidelines or best practices through which a specific requirement from the international legally binding instrument is being or can be implemented. These “implementation examples” include guidance documents and related projects for the implementation of Security Council resolution 1540 (2004) contained in the reports of 1540 Committee for 2008 and 2011 (30, 31), excerpts from the WHO Laboratory biosecurity guidance (32), and provisions of Regulation (EU) No 821/2021 regarding a community export regime of dual-use items (33). The matrices solely contain requirements regarding biosecurity and the wordings of the requirements were simplified. As safety and security aspects are often intertwined, selecting appropriate requirements from the JEE represented a special challenge. Requirements regarding JEE's technical areas zoonotic diseases and food safety were included, as surveillance and response systems for these areas have both safety and security roles.

A comparison of the three legally binding biosecurity instruments demonstrates both differences and similarities. Stemming from the Public Health domain, the International Health Regulations are more extensive than the UNSCR1540 and the BTWC. The IHR contains fields of action that are not represented in the UNSCR1540 and the BTWC. Fields of action that are exclusively stated by the IHR include emergency preparedness and response, risk communication, information sharing, trainings, coordination, communication and advocacy, zoonotic diseases, and food safety. In the fields of action that overlap between the three instruments, IHR is often more extensive than UNSCR1540 and the BTWC.

The differences between the instruments in the field of biosecurity were observed and assuming full implementation of IHR, indicating a maximum score of 5 in all JEE technical areas, it was assessed what else needs to be in place for a country to also comply to UNSCR1540 and BTWC. It can be concluded that in addition to full implementation of the IHR, a comprehensive export control system needs to be in place to also comply to UNSCR1540 and BTWC. Although, it should be noted that the JEE includes some references to export control systems. In JEE's Technical areas 1, 2 and 8 it is indicated in the footnotes that the term “relevant sectors” include, among others, “divisions/activities of other sectors which affect public health, such as ministries of agriculture (quarantine and movement control authority, import/export regulations, disease diagnosis and control financing, zoonosis, veterinary laboratory, etc.) ... trade and/or industry....foreign trade... treasury or finance (customs) …”. (23). Additionally, JEE Technical Area 6 does include a requirement for a mechanism for biosecurity oversight of dual-use research and responsible code of conduct for scientists. However, this requirement does not include an explicit “cross-border” element. An implementation example of comprehensive export control measures is the Regulation of the European Parliament and of the Council No 2021/821 “Setting up a Union regime for the control of exports, brokering, technical assistance, transit and transfer of dual-use items” (33).

Another difference observed in the comparison of IHR, BTWC, and UNSCR1540 is that, IHR and JEE do not deal with non-proliferation. Hence they do not require from countries to enact (criminal) legislation which prohibits and punishes persons or entities who engage in activities related to biological weapons, whereas BTWC and UNSCR1540 do have this requirement. Although IHR and JEE request timely and accurate disease reporting and information sharing, requirements related to cooperative action between states to prevent illicit trafficking in weapons, their means of delivery, and related materials is missing.

Another requirement not mentioned in IHR and JEE that is included in UNSCR1540 deals with participation in other related non-proliferation instruments and mechanisms. UNSCR1540 calls upon states to promote the universal adoption and full implementation of multilateral non-proliferation treaties (e.g., ratification/accession; participation at meetings; delivering of statements; submission of reports).

## Adherence to and Effectiveness of International Instruments

Although there are international instruments in place to prevent outbreaks, regardless of their origin, the COVID-19 pandemic has demonstrated that there is insufficient capacity to prevent and control such a major infectious disease outbreak. This raises questions if the IHR are perhaps deficient and could in fact constrain rather than facilitate rapid action (34). However, the Review Committee on the Functioning of the IHR (2005) during the COVID-19 Response found inadequate IHR implementation and adherence by WHO and its member states (35). States have largely failed to implement the required measures (36). The resulting lack of sufficient capacity to prevent and respond to COVID-19 was demonstrated by the 2021 Global Health Security Index report (8). The report found “Although many countries were able to quickly develop capacities to address COVID-19, all countries remain dangerously unprepared for meeting future epidemic and pandemic threats.”

Global health security would benefit from increased adherence and effectiveness of international biosecurity instruments. Observing differences between legally binding and non-legally binding instruments, it can be assumed that the quality of being “legally binding” is not directly translatable in instrument's effectiveness. An analogy may be drawn to a study in the field of international climate regime that compared the effectiveness of legally vs. non-legally binding instruments. Although formulating an agreement in legally binding terms may lead to stronger commitment, a legal character does not always translate to a higher effectiveness than non-legally binding instruments (37). Non-legally binding instruments can offer a flexible and efficient way to make informal arrangements (38). They may be formulated in a manner that is more technical and therefore “legible” for the experts in the field, opposed to the lofty and often general wordings of conventions and treaties.

Indeed, it is usually so, that non-legally binding instruments are quoted as source of “obligations” for the countries; as the effectiveness of an international instrument, measured by number of countries adhering to it, largely depends on its clarity, value it has for building national capacities and, of course, wide political neutrality. While resolutions, conventions and treaties (legally binding instruments), in their effort to set strategic and global goals, employ ambiguous language trying to encompass a variety of national systems, guidelines and similar tools that aim at building national capacities necessary to reach those strategic goals (non-legally binding instruments) are more precise and attune to the situation on the ground. The former ones are adopted in political forums, while latter are endorsed by expert bodies, which adds to their quality and usefulness. Very often, goal oriented technical discussions and their outcomes diffuse and reduce political frictions that exist on the margins of the central topic, allowing the adopted instruments to be adhered to by the widest audience possible. Therefore, clear non-legally binding instruments, such as national capacity building goals in the field of biosecurity and WHO's Laboratory biosecurity guidance (32), are key for adherence to the legally binding biosecurity instruments. Of course, this depiction is a generalization and does not apply to dichotomies of legally binding and non-legally binding instruments in all areas. In addition, international initiatives such as the Global Health Security Agenda (GHSA), Global Biosecurity Dialog (GBD), and Global Partnership Against the Spread of Weapons and Materials of Mass Destruction (GPWMD) play a major role in building biosecurity capacity and employing international legally binding biosecurity instruments (39).

## International Non-Legally Binding Instruments in the Field of Biosecurity

Non-legally binding instruments are an important addition to legally binding instruments, as they provide practical tools that will help build national capacities. In addition to the legally binding instruments, IHR, BTWC, and UNSCR1540, there are several international non-legally binding instruments, such as guidelines and voluntary arrangements, in the field of biosecurity. Here the most relevant international non-legally binding instruments are highlighted, as these could support adherence and effectiveness of legally binding instruments. In addition, several guidance documents and assessment tools have been developed by national and international organizations, of which many of them have been collected in repositories freely available online (40–42).

In 2002, WHO member states have adopted resolution WHA55.16 on global public health response to natural occurrence, accidental release or deliberate use of biological and chemical agents or radio-nuclear material that affect health (43). This resolution was endorsed in response to the WHO Secretariat's Report on deliberate use of biological and chemical agents to cause harm (A55/20) (44). The resolution urges member states to ensure they have national disease-surveillance plans, to collaborate internationally, and provide mutual support. Furthermore, it encourages member states to treat any deliberate use of biological and chemical agents and radio-nuclear attack to cause harm also as a global public health threat.

The WHO Guidance document on the public health response to biological and chemical weapons was published in 2004 (45). The Guidance describes how biological and chemical agents may endanger public health as well as standard principles of risk management, which are used to outline the steps that member states may take to prepare themselves for the possibility that biological or chemical agents may be deliberately released with the aim of harming their population. It also considers how both national and international law can contribute to preparedness planning, including through established mechanisms for mobilizing international assistance.

The WHO Biorisk management Laboratory biosecurity guidance followed in 2006 (32). This guidance was developed with the aim to integrate the long-known biosafety practices, as described in the WHO Laboratory Biosafety Manual (46), and laboratory biosecurity concept into a comprehensive biorisk management approach. The basic proposition of the Guidance is that the systematic use of appropriate biosafety principles and practices also reduces the risks of valuable biological materials loss, theft or misuse. It provides practical guidance on implementing biosafety and biosecurity.

In 2007, a CEN Workshop adopted a Laboratory Biorisk Management Standard CWA 15793:2008 (47). The document specifies requirements for a biorisk management system that will enable an organization to develop and implement a biorisk policy, establish objectives and processes to achieve the policy commitments and improve its performance. It follows a risk based approach taking in legal requirements and current knowledge and is intended to apply to all types and sizes of organizations and to accommodate diverse geographical, cultural and social conditions. The document is performance oriented, i.e., it describes what needs to be achieved and it is up to the implementing organization to choose the methods and means. CWA 15793:2008 became the backbone of ISO 35001:2019 Biorisk management for laboratories and other related organizations (48). This ISO standard defines a process for identifying, assessing, controlling and monitoring the risks associated with high-risk biological materials.

In 1985, the Australia Group (AG) was established as a voluntary, export-control arrangement through which its participants coordinate their national export controls of chemicals and biological agents as well as related equipment, technologies, and knowledge (49). The Australia Group currently counts forty-three participating countries. The Group issues the Guidelines for Transfers of Sensitive Chemical or Biological Items as well as the Common Control Lists that serve for identification of items whose transfers require license. For the purpose of facilitating effective export controls on Australia Group listed items the United States Government produced the Australia Group Common Control List Handbook (50).

Where the Australia Group is focused on export controls of chemicals and biological agents, the Wassenaar Arrangement on Export Controls for Conventional Arms and Dual-Use Goods and Technologies, has a broader scope (51). The arrangement, formally established in 1996, is a voluntary export control regime whose members exchange information on transfers of conventional weapons and dual-use goods and technologies and currently counts 42 participating states. Among these states are 26 EU Member States, Argentina, Australia, RF, Mexico, South Africa, Turkey, Ukraine, UK, and US. It has been launched with the aim to contribute to regional and international security and stability, by promoting transparency and greater responsibility in transfers of controlled items, thus preventing destabilizing accumulations. Members apply export controls to all items set forth in the List of Dual-Use Goods and Technologies and the Munitions List (52), with the objective of preventing unauthorized transfers or re-transfers of those items.

An overview of all of the described international instruments in the field of biosecurity is presented in Table 1.

**Table 1:** Overview of international instruments in the field of biosecurity.

**Instrument**	**Legal status**	**Domain**	**Scope**
International Health Regulations	Legally binding	Public Health	To prevent, protect against, control, and provide a public health response to the international spread of disease in ways that are commensurate with and restricted to public health risks and that avoid unnecessary interference with international traffic and trade (14)
United Nations Security Council Resolution 1540	Legally binding	Non-proliferation	All states shall refrain from providing any form of support to non-state actors that attempt to develop, acquire, manufacture, possess, transport, transfer or use nuclear, chemical or biological weapons and their means of delivery, in particular for terrorist purposes and shall enforce appropriate legal and regulatory measures against the proliferation of chemical, biological, radiological, and nuclear weapons and their means of delivery (18)
Biological Weapons Convention	Legally binding	Non-proliferation	Prohibits the development, production, acquisition, transfer, stockpiling and use of biological and toxin weapons (17)
World Health Assembly Resolution 55.16	Non-legally binding	Intersection of Public Health and biosecurity	Global public health response to natural occurrence, accidental release or deliberate use of BC agents or RN material (43)
WHO guidance: Public health response to biological and chemical weapons	Non-legally binding	Intersection of Public Health and biosecurity	Outline of steps that member states may take to prepare themselves for the possibility that biological or chemical agents may be deliberately released with the aim of harming their population (45)
WHO Biorisk management—Laboratory biosecurity guidance	Non-legally binding	Intersection of Public Health and biosecurity	Provides practical guidance on implementing biosafety and biosecurity and integrates the long-known biosafety practices and laboratory biosecurity concept into a comprehensive biorisk management approach (32)
ISO 35001:2019 Biorisk management for laboratories and other related organizations	Non-legally binding	Intersection of Public Health and biosecurity	This document defines a process to identify, assess, control, and monitor the risks associated with hazardous biological materials. This document is applicable to any laboratory or other organization that works with, stores, transports, and/or disposes of hazardous biological materials (48)
Australia group guidelines and lists	Non-legally binding	Non-proliferation	Voluntary, export-control arrangement through which its participants coordinate their national export controls of chemicals and biological agents as well as related equipment, technologies, and knowledge (49)
Wassenaar Arrangement	Non-legally binding	Non-proliferation	Voluntary export control for Conventional Arms and Dual-Use Goods and Technologies regime whose members exchange information on transfers of conventional weapons and dual-use goods and technologies, contributing to regional and international security and stability (51)

## Actionable Recommendations

The first step for a country to reach a sustainable level of global health security and to adhere to international legally binding instruments in the field of biosecurity is full implementation of the IHR, as the IHR has the most extensive requirements, in sense of their number as well as scope. Both traits are especially pronounced when the requirements are considered in their “elaborated form” contained in the Joint External Evaluation tool. The implementation of the IHR can be greatly supported by non-legally binding instruments such as WHO Laboratory Biosafety Manual (46) and WHO Biorisk management Laboratory biosecurity guidance (32), providing clear and practical guidance, as well as several other non-legally binding instruments freely available in online repositories (40–42). An example of guidance documents proving to be beneficial to adherence to legally binding biosecurity instruments, is the guidance for stepwise implementation of a National Inventory of Dangerous Pathogens (53). Using this guidance, the government of Uganda successfully implemented a National Inventory of Dangerous Pathogens, which has been recognized by the WHO JEE as contributing to Uganda's developed capacities regarding biosafety and biosecurity (54).

In addition to full implementation of the IHR, countries should implement a comprehensive export control system in order to also comply with UNSCR1540 and BTWC. In this field, countries could make use of best practices from the European Union embodied in its Regulation of the European Parliament and of the Council No 2021/821 “Setting up a Union regime for the control of exports, brokering, technical assistance, transit and transfer of dual-use items” (33) or the World Customs Organization (WCO) Framework of Standards to Secure and Facilitate Global Trade (the WCO SAFE Framework of Standards) (55). States can also benefit from the voluntary, export-control arrangements such as Australia Group and Wassenaar Arrangement, as their tools include a detailed handbook and export control lists. It is recommended for countries to use these precise and hands-on tools to implement a comprehensive export control system and comply with international requirements. Apart from them, further implementation examples are provided in the Supplementary Material.

In addition to a comprehensive export control system, states should enact (criminal) legislation which prohibits and punishes persons or entities who engage in activities related to biological weapons. This legislation should include aspiration of cooperative action between states to prevent illicit trafficking in weapons, their means of delivery, and related materials. National implementation plans and national reports of other countries for UNSCR1540 and BTWC may provide guidance to other countries aiming to implement appropriate legislation.

Furthermore, states should promote the universal adoption and full implementation of multilateral non-proliferation treaties and so achieve their full compliance with international biosecurity legally binding instruments. This can be done by ratification or accession to the treaties, but also by participation in treaty meetings, delivering of statements and submission of country reports. Reports of the 1540 Committee contain shared experiences and related projects for the implementation of UNSCR1540, which cover also this area of implementation.

Lastly, the detailed comparison between the three legally binding international biosecurity instruments demonstrates that the obligations deriving from these instruments have a lot in common, despite the different scopes and domains of these instruments; preparedness and response to natural or accidental outbreaks of infectious diseases vs. a deliberate release with the intension to cause harm. Both domains contain stakeholders in the field of biosecurity, but the domains operate rather independently. As described by Evans et al. global health security could benefit from experimentation in biosecurity governance (56) and biosecurity governance should aim for more connection between the stakeholders concerned.

## Discussion and Conclusion

As demonstrated by the COVID-19 pandemic, the spread of dangerous pathogens represents a serious global health security threat. International instruments from different disciplines address these health and security challenges, setting requirements for states to effectively prevent, detect, and respond to infectious disease outbreaks, either with deliberate or non-deliberate origin. From the detailed comparison between legally binding international biosecurity instruments, it can be concluded that in addition to full implementation of the International Health Regulations, specific export control and non-proliferation measures are necessary to comply with the obligations stemming from other legally-binding international biosecurity instruments. These other instruments also request participation in other related non-proliferation instruments and mechanisms. Adherence to and effectiveness of legally binding biosecurity instruments can be enhanced by clear non-legally binding instruments providing precise guidance and practical implementation examples. These insights highlight the increasing importance of global health diplomacy. Moreover, this paper could facilitate policymakers, civil servants, biosecurity experts, and practitioners to improve both national and international multidisciplinary capacity to protect and defend against biological threats, whether due to natural, accidental, or deliberate causes.

## Author Contributions

IV, MB, DB, and SR contributed to providing an overview and comparison of international biosecurity instruments and reviewed the document. IV wrote and revised the document. MB wrote the Supplementary Material and was a major contributor in writing the manuscript. All authors read and approved the final manuscript.

## Funding

This paper was created as part of EU project, the preparation of a biosecurity toolbox to strengthen European Biosecurity, with financial support from the European Commission—Directorate General Home Affairs (specific contract HOME/2019/ISFP/FW/CBRN/0005 under Framework contract HOME/2014/ISFP/PR/CBRN/0025-Lot 1).

## Conflict of Interest

MB was employed by ENCO Consulting. The remaining authors declare that the research was conducted in the absence of any commercial or financial relationships that could be construed as a potential conflict of interest.

## Publisher's Note

All claims expressed in this article are solely those of the authors and do not necessarily represent those of their affiliated organizations, or those of the publisher, the editors and the reviewers. Any product that may be evaluated in this article, or claim that may be made by its manufacturer, is not guaranteed or endorsed by the publisher.
