# Supplementary material for: Complementarity of International Instruments in the Field of Biosecurity
Source: Front Public Health. 2022 May 27;10:894389. doi: 10.3389/fpubh.2022.894389 (PMC9195852; doi:10.3389/fpubh.2022.894389)
Supplement: Supplementary file 1 [file Data_Sheet_1.PDF]

COMPLEMENTARITY OF INTERNATIONAL INSTRUMENTS IN THE FIELD OF BIOSECURITY

SUPPLEMENTARY MATERIAL

Contents

Matrices – obligations stemming from UNSC Resolution 1540, BTWC and IHR 2
- Table S1: Prevention 7
- Table S2: Prohibitions & Penalties 71
- Table S3: Detection 74
- Table S4: Response 78
- Table S5: International cooperation, participation in related legally binding instruments, mechanisms or IOS 84

Matrices – obligations stemming from UNSC Resolution 1540, BTWC and IHR

CLUSTERS

Taking into account different methods for grouping of obligations stemming from UNSC Resolution 1540, BTWC and IHR (2005) and the fact that the Joint External Evaluation Tool for IHR has the most extensive number of requirements organised in 4 segments (prevent, detect, respond, and IHR related hazards and points of entry), Matrices were made for the following fields of action (clusters), making a combination of the JEE's groups and similar tasks from other two instruments:

1. Prevention;
2. Prohibition and penalties;
3. Detection;
4. Response;
5. International cooperation, participation in related legally binding instruments, mechanisms or IOs.

In its substance the **preventive actions cluster** (1) is closely linked to the one on **prohibitions and penalties** (2), as proscribing certain activities and setting appropriate civil and criminal penalties for them acts as a deterrent, *i.e.* can be considered as a preventive measure.

Provisions/articles requiring from states themselves not to undertake certain activities are considered to be translated through national legal, administrative, judicial or any other measures which prohibits undertaking of such activities to everyone (i.e. including state authorities and their representatives). Hence, such provisions/articles of international instruments are included in the **section on prohibitions and penalties** (2).

SPECIFIC REQUIREMENTS

Specific requirements included in the Matrices are compiled not only by using texts of UNSC Resolution 1540, BTWC and IHR (2005), but also additional documents that are considered to represent authentic "interpretation" tools for them. Thus, for the UNSC Resolution the Approved 1540 Committee Matrix [1] provided wordings for individual requirements, for the Biological Convention the wordings were sourced also from the Additional Agreements and Understandings and the Guide to Participating in the Confidence-Building Measures, while for the IHR (2005) the Joint External Evaluation Tool served this purpose.

Meaning of "account for", "secure" and "physical protection" – specific requirements stemming from operative paragraph 3 of Resolution 1540

In its operative paragraph 3 Resolution 1540 requires from states to:

a) develop and maintain appropriate effective measures **to account for and secure** such items in production, use, storage or transport, and
b) develop and maintain appropriate effective **physical protection measures**".

These requirements were then further atomised by 1540 Committee within 1540 Matrix into the following specific tasks:

1. Measures to <u>**account**</u> for production,
2. Measures to account for use,

[1] "Since its adoption in early 2005, the 1540 Matrix has functioned as the primary method used by the 1540 Committee to organize information about implementation of UN Security Council resolution 1540 by Member States.... **The matrix has fields representing the requirements of the resolution** alongside which are the measures that States have taken in respect of these requirements", Approved 1540 Committee Matrix, available at https://www.un.org/en/sc/1540/national-implementation/1540-matrices.shtml, retrieved 16 September 2019

3. Measures to account for storage,
4. Measures to account for transport,
5. Measures to **secure** production,
6. Measures to secure use,
7. Measures to secure storage,
8. Measures to secure transport,
9. **Physical protection measures**,
10. **Personnel Reliability**,
11. **Licensing system.**

However, the exact definitions and the scope of these specific tasks could not be determined based on any of the documents which 1540 Committee, the creator of 1540 Matrix, keeps available for the Member States on its Homepage.

With the aim to still keep 1540 Matrix integrated as much as possible into the presented Matrices (on obligations stemming from UNSC Resolution 1540, BTWC and IHR) the mentioned eleven tasks were regrouped by the authors as shown in the table below.

**CLUSTER**	**SPECIFIC REQUIREMENTS**
	**UNSC Res. 1540**
**PREVENTION**	
ADEQUATE LEGAL, POLICY FRAMEWORK, FINANCING AND HUMAN RESOURCES<br>⇔<br>COHERENT **PHYSICAL PROTECTION REGIME**	***Para 3, subpara (b)* →**<br>*Establishing coherent physical protection regime, including legislative and regulatory framework governing the physical protection of sensitive materials and associated facilities as well as the institutions and organizations within the State responsible for ensuring implementation of the relevant legislation*
	Physical protection **regime**
	Licensing system
**ACCOUNT AND SECURE**	***Para 3, subpara (a)* →**<br>*Measures to establish domestic controls to prevent the proliferation of biological weapons and their means of delivery; controls over related materials*
	Personnel reliability
	Measures to account for production, use, storage
	Measures to account for transport
	Measures to secure production, use, storage
	Measures to secure transport

As it can be seen 1540 Resolution's "physical protection **measures**" were converted into "physical protection **regime**". This was done with the aim to make a distinction between "physical protection" obligations and obligations "to secure" materials, which are closely interlinked. Untangling these terms and their underlying obligations was done based on the analysis of the IAEA nuclear security publications[2] and WHO Laboratory biosecurity guidance (2006).

Physical Protection Measures ⇔ Physical Protection Regime

According to the latest IAEA Security Series Glossary, "physical protection" represents a set of measures, including structural, technical and administrative protective measures, taken to prevent an adversary from achieving an undesirable consequence, such as radiological sabotage, or the unauthorized removal of nuclear or other radioactive material in use, storage or transport, and to mitigate or minimize the consequences if the adversary initiates such a malicious act[3]. WHO Laboratory biosecurity guidance defines a similar term "physical biosecurity"[4] as a set "of engineering, structural and security personnel elements, intended to select, control and document access to laboratories and to the materials they contain, and to limit improper removal of valuable biological materials and equipment. Access controls are used to limit access to restricted areas to individuals who have proper authorization and to keep track of traffic in and out of these areas". It can be concluded that in this context actions intended at securing[5] sensitive materials are types of physical protection measures. In an attempt to overcome this tautology, the requirement for physical protection measures was converted into a wider requirement for establishing a state's physical protection regime. Such a regime implies the establishment of a legislative and regulatory framework governing the physical protection of sensitive materials in their production, use, storage and transport as well as creation institutions and organizations within the State responsible for ensuring implementation of the relevant legislation. Apart from materials, the regime also protects associated facilities. The requirement to establish a regime goes above the mere sum of measures for securing materials. It entails that these measures are set in national laws and regulations, that there are central authorities in charge for ensuring the implementation of the applicable legal framework, including through monitoring and inspections[6]. Understood like this the physical protection regime encompasses elements included, among others, in the JEE's Technical Area 1 and 11, i.e. National legislation, policy and financing and Human resources respectively. The physical protection regime also involves a coherent system of response to a malicious act with the aim to minimise or mitigate its consequences. In the biosecurity field additionally it covers policy and legal actions intended to minimise threat through measures such as consolidation of dangerous pathogens and toxins into a minimum number of facilities.

With regard to the meaning of the term **"to account for"** valuable biological materials, within the scope of this paper, it represents a system of **procedural measures** necessary to establish and **track material inventories**, **control access**

---

[2] As the nuclear field has the advantage of having a focal international organisation for the development of harmonised glossaries and more importantly having in mind that the wording "physical protection" is coming from the nuclear field.

[3] "**Physical protection:** 1. The *nuclear security* of *nuclear material* and *nuclear facilities*. A footnote in Ref. [13R] effectively defines *physical protection* in this way. Hence, when the context is clearly *nuclear material* and *nuclear facilities*, *physical protection* and *nuclear security* may be considered synonymous. 2. Measures (including structural, technical and administrative protective measures) taken to prevent an *adversary* from achieving an undesirable consequence (such as radiological *sabotage*, or the *unauthorized removal* of *nuclear or other radioactive material* in use, storage or transport) and to mitigate or minimize the consequences if the *adversary* initiates such a *malicious act*. [16T]

Physical protection measures: The personnel, procedures, and equipment that constitute a *physical protection system*. [13R, 26G]

Physical protection regime: A State's regime including: the legislative and regulatory framework governing the physical protection of *nuclear material* and *nuclear facilities*; the institutions and organizations within the State responsible for ensuring implementation of the legislative and regulatory framework; facility and transport *physical protection systems*. [13R, 26G]

Physical protection system: An integrated set of *physical protection measures* intended to prevent the completion of a *malicious act*." IAEA Nuclear Security Series Glossary 2015, available at https://www-ns.iaea.org/downloads/security/nuclear-security-series-glossary-v1-3.pdf, retrieved on 8 October 2019

[4] "Physical Security means the assurance of safety from physical intrusion. It comprises of engineering, structural and security personnel elements intended to select, control and document access to laboratories and to the materials they contain, and to limit improper removal of valuable biological materials and equipment", National Laboratory Biosecurity Assessment and Monitoring Checklist (In the Framework of the Biological Weapons Convention), Science and Technology Research Institute for Defence in cooperation with UNODA and RIVM, 2018

[5] The term "secure" within the scope of this document is understood as putting **into place structural and technical measures**, including **security personnel**, with the aim prevent access to valuable biological materials.

[6] In the absence of a national regime or in case of its incompleteness laboratory managers may still implement specific physical protection measures of their choice. This kind of situations represent a clear example for distinction between a PP regime and PP measures.

to and **detect loss** or diversion of agents including assignment of responsibility to the **individuals who provide oversight** and are held responsible for valuable biological materials.

Meaning of shadings and fonts

With regard to the meanings of shadings and fonts in the Matrices, it should be noted that **table cells shaded in light grey** with italic text in them followed by an arrow (→) provide **a description** of the main obligation stemming from a paragraph/article and **represent a chapeau** for the specific requirements.

Example:

Para 3, subpara (a) (b) *→ Measures to establish domestic controls to prevent the proliferation of biological weapons and their means of delivery[7]; controls over related materials[8]*	→ A **description** of the main obligation stemming from a paragraph/article
Personnel Reliability	
Measures to account for production, use, storage	→ **Specific requirements**
Etc.	

Each **new main obligation** stemming from a paragraph/article and specific requirement arising from it are demarcated with **dark grey rows** coming before and after it.

Example:

	← **demarcation**
Para 3, subpara (a) (b) *→ Measures to establish domestic controls to prevent the proliferation of biological weapons and their means of delivery[9]; controls over related materials[10]*	
Measures to account for production, use, storage	
Measures to account for transport	
	← **demarcation**
Article II *→ To destroy* ***or divert*** *to peaceful purposes all agents, toxins, weapons, equipment and means of delivery specified in Article I prior to joining the Convention.*	
The Sixth and Seventh Review Conferences emphasised that states must take all necessary **safety and security provisions to protect** populations and the environment **when carrying out such destruction and/or diversion**. The Seventh Review Conference emphasised that these measures should protect human populations and the environment, including animals and plants.	

[7] UNSC Resolution 1540: "Means of delivery: missiles, rockets and other unmanned systems capable of delivering nuclear, chemical, or biological weapons, that are specially designed for such use."

[8] UNSC Resolution 1540: "Related materials: materials, equipment and technology covered by relevant multilateral treaties and arrangements, or included on national control lists, which could be used for the design, development, production or use of nuclear, chemical and biological weapons and their means of delivery."

[9] UNSC Resolution 1540: "Means of delivery: missiles, rockets and other unmanned systems capable of delivering nuclear, chemical, or biological weapons, that are specially designed for such use."

[10] UNSC Resolution 1540: "Related materials: materials, equipment and technology covered by relevant multilateral treaties and arrangements, or included on national control lists, which could be used for the design, development, production or use of nuclear, chemical and biological weapons and their means of delivery."

Division within the Matrices

Each matrix is split into columns that include specific requirements stemming from 1540 Resolution, BTWC and JEE. **The last column on implementation** includes references to or extracts from international instruments, guidelines or best practices through which a specific requirement from the international legally binding instrument is being/can be implemented. These "implementation examples" include:

- experience shared and related projects for the implementation of Security Council resolution 1540 (2004) contained in the **reports of 1540 Committee for 2008 and 2011**[11];
- excerpts from the **WHO Laboratory biosecurity guidance**;
- provisions of **Regulation (EU) 2021/821 of the European Parliament and of the Council of 20 May 2021 setting up a Union regime for the control of exports, brokering, technical assistance, transit and transfer of dual-use items.**

[11] Experience shared and related projects for the implementation of Security Council resolution 1540 (2004): update to annex XVII of the previous report of the Committee established pursuant to resolution 1540 (2004) to the Security Council (S/2008/493) (Annex XVI of 2011 Committee Report S/2011/579) and Experience shared for the implementation of Security Council resolution 1540 (2004) (Annex XVII of 2008 Committee Report S/2008/493), both available at https://www.un.org/en/sc/1540/resolutions-committee-reports-and-SC-briefings/committee-reports-to-the-security-council.shtml (last accessed on 3 February 2022)

Table S1: Prevention

CLUSTER	SPECIFIC REQUIREMENTS			IMPLEMENTATION
	**UNSC Res. 1540**	**BTWC**	**IHR (2005)**	
**PREVENTION**				
ADEQUATE LEGAL, POLICY FRAMEWORK, FINANCING AND HUMAN RESOURCES<br><br>⇔<br><br>COHERENT PHYSICAL PROTECTION REGIME[12]	***Para 3, subpara (b) →***<br><br>*Establishing coherent physical protection regime, including legislative and regulatory framework governing the physical protection of sensitive materials and associated facilities as well as the institutions and organizations within the State responsible for ensuring implementation of the relevant legislation*	***Article III*** → *Not to transfer, or in any way assist, encourage or induce anyone else to acquire or retain biological weapons.*<br><br>***Article IV*** → *To take any national measures necessary to implement the provisions of the BTWC domestically*	***JEE Technical area 1: National legislation, policy and financing***<br>→ *The JEE considers the adequate legal framework governing public health surveillance and response to underpin the implementation of all obligations and rights endorsed by the IHR (including those relevant for biosecurity) and to pertain to the "prevention" cluster[13]. Adequate policy and adequate financing are part of the same JEE technical area.*	

[12] See section 5.1.1. Meaning of "account for", "secure" and "physical protection"

[13] The Joint External Evaluation Tool divides 19 technical areas into four clusters: prevent, detect, respond and IHR related hazards and points of entry.

CLUSTER	SPECIFIC REQUIREMENTS			IMPLEMENTATION
	UNSC Res. 1540	BTWC	IHR (2005)	
			JEE Technical area 11: Human resources[14]<br>→ *Prevention, detection and response activities (including health promotion, occupational health safety and security, and appropriate care of those affected) need to be conducted effectively and sustainably by a fully competent, coordinated, evaluated and occupationally diverse multisectoral workforce.*<br><br>***JEE Technical Area 6*: (Biosafety and) Biosecurity**[15]<br>→ *A whole-of-government multisectoral national (biosafety and) biosecurity system (for all sectors including human, animal and agriculture*	

[14] Technical area Human resources is assigned to the group "Detection" within the JEE Tool; however, the author of the Matrix considers human resources to be a precondition for a functioning system, the same way legislation and finances are.

[15] "Laboratory biosecurity describes the protection, control and accountability for valuable biological materials within laboratories as well as information related to these materials and dual-use research, in order to prevent their unauthorized access, loss, theft, misuse, diversion or intentional release", Joint external evaluation tool: International Health Regulations (2005), second edition. Geneva: World Health Organization; 2018. License: CC BY-NC-SA 3.0 IGO

CLUSTER	SPECIFIC REQUIREMENTS			IMPLEMENTATION
	UNSC Res. 1540	BTWC	IHR (2005)	
			facilities) with dangerous pathogens[16] identified, held, secured and monitored in a minimal number of facilities according to best practices; biological risk management training and educational outreach conducted to promote a shared culture of responsibility[17], reduce dual-use risks, mitigate biological proliferation and deliberate use threats, and ensure safe transfer of biological agents; and country- specific (biosafety and) biosecurity legislation, laboratory licensing and pathogen control measures in place as appropriate.	

[16] "Dangerous pathogens and toxins – The informal Australia Group provides a List of human and animal pathogens and toxins for export control (http://www.australiagroup.net/en/human_animal_pathogens.html, accessed 23 November 2017)", *Ibidem*
[17] "Responsible life sciences research for global health security: a guidance document. WHO/HSE/GAR/BDP/2010.2. Geneva: World Health organization; 2010 (http://www.who.int/csr/resources/publications/HSE_GAR_BDP_2010_2/en/ (accessed 21 November 2017)", *Ibidem*

CLUSTER	SPECIFIC REQUIREMENTS			IMPLEMENTATION
	**UNSC Res. 1540**	**BTWC**	**IHR (2005)**	
	Physical Protection regime	**Article III**<br>The Sixth Review Conference called for appropriate measures by all States Parties to ensure that biological agents and toxins relevant to the Convention are protected and safeguarded, including through measures to control access to and handling of such agents and toxins.<br><br>**Article IV**<br>The Second, Third and Fourth Review Conferences noted *the* importance of... legislation regarding the physical protection of laboratories and facilities to prevent unauthorized access to and removal of microbial or other biological agents, or toxins	**JEE Technical Area 1**<br>Legislation including laws, regulations, administrative requirements, policies or other government instruments, proven to be adequate in all (biosecurity) relevant sectors to support IHR implementation.<br><br>**JEE Technical Area 1**<br>Adequate finances available to enable efficient and effective IHR implementation and response to all public health emergencies.<br><br>**JEE Technical area 11**<br>A trained health workforce that includes nurses and midwives, physicians, public health and environmental specialists, social scientists, laboratory scientists/technicians, biostatisticians, IT specialists and biomedical technicians.	

CLUSTER	SPECIFIC REQUIREMENTS			IMPLEMENTATION
	UNSC Res. 1540	BTWC	IHR (2005)	
			**JEE Technical area 11** Existence of a corresponding workforce in the animal sector of veterinarians, para-veterinarians, animal health professionals, epidemiologists, IT specialists, and others.<br><br>**JEE Technical Area 6** On a national level – establishment of an **agreed List** of "dangerous pathogens and toxins".<br><br>**JEE Technical Area 6** On a national level - monitoring and maintaining **a national record** of where and in which facilities dangerous pathogens and toxins are housed is led.<br><br>**JEE Technical Area 6** On a national level - Consolidation of dangerous pathogens and toxins into a **minimum**	

CLUSTER	SPECIFIC REQUIREMENTS			IMPLEMENTATION
	UNSC Res. 1540	BTWC	IHR (2005)	
			**number of facilities** is achieved.<br><br>JEE Technical Area 6<br>There is a framework to document, report, investigate and address any incidents at the facility and national levels.<br><br>JEE Technical Area 6<br>Mechanisms for oversight and enforcement are in place.	
	Licensing and/or registration of:<br>a) installations, facilities, persons, entities<br>b) use, handling of materials	**CBM Guide to Participating in the CBMs**<br><br>"Scope of Form E - Implementation is understood to include all measures that help to meet the aims and objectives of the Convention....Form E addresses: ...<br><br>3. Legislation and regulation relevant to Article IV of the Convention (prohibition and prevention) and to agreements by Review Conferences for protecting and safeguarding biological	JEE Technical Area 6<br>Enacted comprehensive national biosecurity regulatory framework, including laboratory licensing with the security measures as a licensing condition.	

CLUSTER	SPECIFIC REQUIREMENTS			IMPLEMENTATION
	UNSC Res. 1540	BTWC	IHR (2005)	
		agents and toxins relevant to the Convention; for example, legislation, regulations and other measures relating to biosafety and biosecurity. Examples of such measures might include:<br>– Introducing a system authorizing the possession of specific biological agents and toxins, including the licensing of personnel;<br>– Authorizing the use of specific biological agents and toxins;<br>– Licensing work on the genetic modification of biological agents;<br>– Ensuring the traceability of biological agents and toxins;<br>– Introducing a system authorizing the transport of		

CLUSTER	SPECIFIC REQUIREMENTS			IMPLEMENTATION
	UNSC Res. 1540	BTWC	IHR (2005)	
		biological agents and toxins on national territory;<br>– Introducing national systems for the inspection of facilities possessing specific biological agents and toxins;<br>– Ensuring the physical protection of facilities possessing biological agents and toxins;<br>– Introducing a mandatory system for the notification of the loss or theft of biological agents and toxins;...”		
COORDINATION, COMMUNICATION AND ADVOCACY			***JEE Technical Area 2: IHR Coordination, communication and advocacy***<br>*→A mechanism for multisectoral/multidisciplinary coordination,*	

CLUSTER	SPECIFIC REQUIREMENTS			IMPLEMENTATION
	UNSC Res. 1540	BTWC	IHR (2005)	
			communication and partnerships to detect, assess and respond to any public health event or risk.<br><br>***JEE Technical Area 14: Linking public health and security authorities →*** *Development and implementation of a MoU or other similar framework outlining roles, responsibilities and best practices for sharing relevant information between and among appropriate human and animal health, law enforcement and defence personnel, and validation of the MoU through periodic exercises and simulations.*	
			**JEE Technical Area 2** Establishment of a functional multisectoral and multidisciplinary mechanism for **the coordination and integration** of relevant	

CLUSTER	SPECIFIC REQUIREMENTS			IMPLEMENTATION
	**UNSC Res. 1540**	**BTWC**	**IHR (2005)**	
			sectors in the implementation of IHR and to respond to any public health events (e.g. interagency bodies, multisectoral coordination committees, action/response plans, SOPs, National Action Plan for Health Security, etc.)	
			**JEE Technical Area 2**<br>Regular **testing** of the mechanism for communication and coordination through **exercises** and subsequent **improvement** of arrangements and procedures.	
			**JEE Technical Area 14**<br>SOPs or agreements are in place for coordination of a joint response to public health and other emergencies at official locations, such as points of entry where both public health and security authorities have	**WHO Laboratory biosecurity guidance, Chapter 6. Laboratory biosecurity programme, 6.1 Laboratory biosecurity risk assessment**<br>In the context of a biosecurity risk assessment, security and intelligence forces play the fundamental role of complementing the biosafety risk assessments performed by the laboratory management with local threat assessments. Collaboration between these

CLUSTER	SPECIFIC REQUIREMENTS			IMPLEMENTATION
	UNSC Res. 1540	BTWC	IHR (2005)	
			operational safety and health security responsibilities.	different stakeholders and proactive clarification of their roles, responsibilities and authorities should help in case of emergencies, where first-responders need the appropriate information, knowledge and skills to provide the most appropriate interventions<br><br>***6.2 Responsibility for VBM***<br>Facilities that handle dangerous pathogens and toxins should ensure that all emergency response personnel, including local law enforcement, are aware of the safety issues on-site and the procedures to be followed if an incident occurs.
			**JEE Technical Area 14**<br>There is a here a functional mechanism for collaboration and timely and systematic information exchange between public health and law enforcement agencies in case of deliberate and/or accidental events.	In addition to above:<br><br>**Chapter 2. Laboratory biosecurity as a complement to laboratory biosafety**<br>Likewise, institutional laboratory biosecurity protocols should include how to handle breaches or near-breaches in laboratory biosecurity including:<br>1. incident notification,<br>2. reporting protocols,<br>3. investigation reports,<br>4. recommendations and remedies, and<br>5. oversight and guidance through the Biosafety Committee.

CLUSTER	SPECIFIC REQUIREMENTS			IMPLEMENTATION
	UNSC Res. 1540	BTWC	IHR (2005)	
				...The involvement, roles and responsibilities of public health and security authorities in the event of a security breach should also be clearly defined.
ACCOUNT AND SECURE[18]	***Para 3, subpara (a)*** → <br>*Measures to establish domestic controls to prevent the proliferation of biological weapons and their means of delivery[19]; controls over related materials[20].*	***Article III*** → *Not to transfer, or in any way assist, encourage or induce anyone else to acquire or retain biological weapons.* <br>***Article IV*** → *To take any national measures necessary to implement the provisions of the BTWC domestically*	***JEE Technical Area 6*: *(Biosafety and) Biosecurity*** → *A whole-of-government multisectoral national (biosafety and) biosecurity system (for all sectors including human, animal and agriculture facilities) with dangerous pathogens[21] identified, held, secured and monitored in a minimal number of facilities according to best practices; biological risk*	Tools facilitating implementation of this specific requirement stemming from Resolution 1540 mentioned in Annex XVII of the 1540 Committee's Report S/**2008**/493: - The Public health response to biological and chemical weapons: WHO guidance 2004 (especially Annex 5 Precautions against the sabotage of drinking-water, food, and other products); - WHO Laboratory Bio-safety Manual 2004 (includes guidance on laboratory biosecurity and regulations for the transport of infectious substances)[23];

[18] See section 5.1.1. Meaning of "account for", "secure" and "physical protection"

[19] UNSC Resolution 1540: "Means of delivery: missiles, rockets and other unmanned systems capable of delivering nuclear, chemical, or biological weapons, that are specially designed for such use."

[20] UNSC Resolution 1540: "Related materials: materials, equipment and technology covered by relevant multilateral treaties and arrangements, or included on national control lists, which could be used for the design, development, production or use of nuclear, chemical and biological weapons and their means of delivery."

[21] "Dangerous pathogens and toxins – The informal Australia Group provides a List of human and animal pathogens and toxins for export control (http://www.australiagroup.net/en/human_animal_pathogens.html, accessed 23 November 2017)", *Ibidem*

[23] Quoted from the 1540 Committee's Report S/2008/493 Annex XVII, published in 2008. The updated WHO Laboratory Bio-safety Manual 2020 fourth edition is available at https://www.who.int/publications/i/item/9789240011311?sequence=1&isAllowed=y (last accessed on 11 February 2022)

CLUSTER	SPECIFIC REQUIREMENTS			IMPLEMENTATION
	**UNSC Res. 1540**	**BTWC**	**IHR (2005)**	
			management training and educational outreach conducted to promote a shared culture of responsibility[22], reduce dual-use risks, mitigate biological proliferation and deliberate use threats, and ensure safe transfer of biological agents; and country- specific (biosafety and) biosecurity legislation, laboratory licensing and pathogen control measures in place as appropriate.	- WHO Preparedness for the Deliberate Use of Biological Agents 2002.<br>- WHO Guidelines for the Surveillance and Control of Anthrax in Humans and Animals,<br>- WHO Guidelines on Tularaemia<br>Tools facilitating implementation of this specific requirement stemming from Resolution 1540 mentioned in Annex XVII of the 1540 Committee's Report S/**2011**/579:<br>- WHO Terrorist threats to food: guidance for establishing and strengthening prevention and response systems
	Personnel Reliability[24]	**CBM Guide to Participating in the CBMs**<br>"Scope of Form E - Implementation is understood to include all measures that help to meet	JEE Technical Area 6<br>A mechanism to determine which personnel are authorized to access pathogens of	**WHO Laboratory biosecurity guidance, Chapter 6. Laboratory biosecurity programme, 6.3 Elements of a laboratory biosecurity plan, Personnel management**<br>Personnel management procedures should define the roles, responsibilities and

[22] "Responsible life sciences research for global health security: a guidance document. WHO/HSE/GAR/BDP/2010.2. Geneva: World Health organization; 2010 (http://www.who.int/csr/resources/publications/HSE_GAR_BDP_2010_2/en/ (accessed 21 November 2017)", *Ibidem*

[24] Personnel reliability refers to programs intended to reduce the prospect that researchers who are permitted to work on hazardous biological agents might misuse that access (screening procedures, risk assessments, etc.). In the National Laboratory Biosecurity Assessment and Monitoring Checklist (In the Framework of the Biological Weapons Convention) it is defined as policies and procedures that define roles, responsibilities and authorities of laboratory personnel who need to handle, use, store and transfer and/or transport valuable biological materials, and the manner in the organization ensures that individuals appropriate for the position they hold.

CLUSTER	SPECIFIC REQUIREMENTS			IMPLEMENTATION
	UNSC Res. 1540	BTWC	IHR (2005)	
		the aims and objectives of the Convention....Form E addresses: ...<br>3. Legislation and regulation relevant to Article IV of the Convention (prohibition and prevention) and to agreements by Review Conferences for protecting and safeguarding biological agents and toxins relevant to the Convention; for example, legislation, regulations and other measures relating to biosafety and biosecurity. Examples of such measures might include:<br>- Introducing a system authorizing the possession of specific biological agents and toxins, **including the licensing of personnel;**	security concern is in place.	authorities of laboratory personnel who need to handle, use, store, transfer and/or transport VBM, and the manner in which the organization ensures that individuals are appropriate for the positions they hold.
	Measures to account for production, use, storage	**Article III**<br>The Sixth Review Conference called for appropriate measures by all	**JEE Technical Area 6**<br>On the level of a facility/entity -Biosecurity	**WHO Laboratory biosecurity guidance, Chapter 5. Countering biorisks, 5.1 Accountability for VBM**

CLUSTER	SPECIFIC REQUIREMENTS			IMPLEMENTATION
	UNSC Res. 1540	BTWC	IHR (2005)	
		States Parties to ensure that biological agents and toxins relevant to the Convention are protected and safeguarded, including through measures to control access to and handling of such agents and toxins.<br><br>**Article IV**<br>The Sixth and Seventh Review Conferences called upon States Parties to adopt, in accordance with their constitutional processes, legislative, administrative, judicial and other measures, including penal legislation, designed to... ensure the safety and security of microbial or other biological agents or toxins in laboratories, facilities, and during transportation, to prevent unauthorized access to and removal of such agents or toxins	**risks have been assessed** and categorized.<br><br>**JEE Technical Area 6**<br>On the level of a facility/entity –Monitoring and **maintaining an updated record and inventory** of pathogens within facilities that store or process dangerous pathogens and toxins.<br><br>**JEE Technical Area 6**<br>On the level of a facility/entity -**Access to sensitive information** (such as inventory of agents and toxins) is controlled by adequate policies and procedures.<br><br>**JEE Technical Area 6**<br>On the level of a facility/entity –Biosecurity control measures are described in an action plan.<br><br>**JEE Technical Area 6**<br><br>On the level of a facility/entity –Roles and	Specific accountability procedures for VBM require the establishment of effective control procedures to track and document the inventory, use, manipulation, development, production, transfer and destruction of these materials. The objective of these procedures is to know which materials exist in a laboratory, where they are located, and who has responsibility for them at any given point in time. To achieve this, management should define:<br>1. which materials (or forms of materials) are subject to material accountability measures;<br>2. which records should be kept, by whom, where, in what form and for how long;<br>3. who has access to the records and how access is documented;<br>4. how to manage the materials through operating procedures associated with them (e.g. where they can be stored and used, how they are identified, how inventory is maintained and regularly reviewed, and how destruction is confirmed and documented);

CLUSTER	SPECIFIC REQUIREMENTS			IMPLEMENTATION
	**UNSC Res. 1540**	**BTWC**	**IHR (2005)**	
			responsibilities related to biosecurity management are defined and documented (security manager).<br><br>[JEE Technical Area 6](#)<br>Monitoring and maintaining an updated record and inventory of pathogens within facilities that store or process dangerous pathogens and toxins.	5. which accountability procedures will be used (e.g. manual log book, electronic tables, etc.);<br>6. which documentation/reports are required;<br>7. who has responsibility for keeping track of VBM;<br>8. who should clear and approve the planned experiments and the procedures to be followed;<br>9. who should be informed of and review the planned transfer of VBMs to another laboratory.<br><br>Accountability also means ensuring that materials are properly safeguarded. A person(s) with expert knowledge of the material in use and its storage should be accountable. Any anomalies seen by the employee should be promptly reported to the laboratory manager.
	Measures to account for transport	[Article IV](#)<br><br>The Sixth and Seventh Review Conferences called upon States Parties to	[JEE Technical Area 6](#)<br><br>National legislation for the transportation of dangerous goods[25],	**WHO Laboratory biosecurity guidance, Chapter 5. Countering biorisks, 5.3 Legitimate research, codes of conduct and codes of practice**

[25] International standards with regard to the transport of dangerous goods stipulate also measures to account for those materials while being transported.

CLUSTER	SPECIFIC REQUIREMENTS			IMPLEMENTATION
	UNSC Res. 1540	BTWC	IHR (2005)	
		adopt, in accordance with their constitutional processes, legislative, administrative, judicial and other measures, including penal legislation, designed to... ensure the safety and security of microbial or other biological agents or toxins in laboratories, facilities, **and during transportation**, to prevent unauthorized access to and removal of such agents or toxins	including pathogens is in place.	Transport security endeavours to provide a measure of security during the movement of biological materials outside of the access-controlled areas in which they are kept until they arrive at their destination. Transport security applies to biological materials within a single institution and between institutions. Internal material transport security includes **reasonable documentation, accountability** and control over VBM moving between secured areas of a facility as well as internal delivery associated with shipping and receiving processes. External transport security should ensure **appropriate authorization** and communication between facilities before, during and after external transport, which may involve the commercial transportation system. The recommendations of the United Nations Model Regulations for the Transport of Dangerous Goods (*23*), providing countries with a framework for the development of national and international transport regulations include provisions addressing the security of dangerous goods, including infectious substances, during transport by all modes.
	Measures to secure production, use, storage	**Article III**<br><br>The Sixth Review Conference called for	**JEE Technical Area 6**<br><br>Appropriate security measures to minimize	**WHO Laboratory biosecurity guidance, Chapter 6. Laboratory biosecurity**

CLUSTER	SPECIFIC REQUIREMENTS			IMPLEMENTATION
	UNSC Res. 1540	BTWC	IHR (2005)	
		appropriate measures by all States Parties to ensure that biological agents and toxins relevant to the Convention are protected and safeguarded, including through measures to control access to and handling of such agents and toxins.<br><br>**Article IV**<br><br>The Sixth and Seventh Review Conferences called upon States Parties to adopt, in accordance with their constitutional processes, legislative, administrative, judicial and other measures, including penal legislation, designed to... ensure the safety and security of microbial or other biological agents or toxins in laboratories, facilities, and during transportation, to prevent unauthorized access to and removal of such agents or toxins	potential inappropriate removal or release of biological agents are in place	**programme, 6.3 Elements of a laboratory biosecurity plan, Physical biosecurity**<br><br>Physical biosecurity, comprised of engineering, structural and security personnel elements, is intended to select, control and document access to laboratories and to the materials they contain, and to limit improper removal of VBM and equipment. Access controls are used to limit access to restricted areas to individuals who have proper authorization and to keep track of traffic in and out of these areas.

CLUSTER	SPECIFIC REQUIREMENTS			IMPLEMENTATION
	**UNSC Res. 1540**	**BTWC**	**IHR (2005)**	
	Measures to secure transport	**Article IV**<br><br>The Sixth and Seventh Review Conferences called upon States Parties to adopt, in accordance with their constitutional processes, legislative, administrative, judicial and other measures, including penal legislation, designed to... ensure the safety and security of microbial or other biological agents or toxins in laboratories, facilities, **and during transportatio**n, to prevent unauthorized access to and removal of such agents or toxins	**JEE Technical Area 6**<br><br>National regulations are in place and up-to-date for the transport of infectious substances (Categories A and B).<br><br>Procedures for a secure transport of culture, specimens, samples and other contaminated materials are established and followed.	Tools facilitating implementation of this specific requirement stemming from Resolution 1540 mentioned in Annex XVII of the 1540 Committee's Report S/**2008**/493:<br><br>- UNECE Recommendations on the Transport of Dangerous Goods - Model Regulations;<br>- European Agreement concerning the International Carriage of Dangerous Goods by Road (ADR);<br>- European Agreement concerning the International Carriage of Dangerous Goods by Inland Waterways (ADN);<br>- OTIF Regulations Concerning the International Carriage of Dangerous Goods by Rail (RID);<br>- Maritime Dangerous Goods (IMDG) Code;<br>- IMO Revised Recommendations on the Safe Transport of Dangerous Cargoes and Related Activities in Port Areas<br>- IMO International Ship and Port Facility Security (ISPS) Code.<br>- Annex 18 to the Convention on International Civil Aviation covers the safe transport of dangerous goods by

CLUSTER	SPECIFIC REQUIREMENTS			IMPLEMENTATION
	UNSC Res. 1540	BTWC	IHR (2005)	
				air and includes a requirement that such transport follow the Technical Instructions for the Safe Transport of Dangerous Goods by Air.<br>**WHO Laboratory biosecurity guidance, Chapter 5. Countering biorisks, 5.3 Legitimate research, codes of conduct and codes of practice**<br>Transport security endeavours to provide a measure of security during the movement of biological materials outside of the access-controlled areas in which they are kept until they arrive at their destination. Transport security applies to biological materials within a single institution and between institutions. Internal material transport security includes reasonable documentation, accountability and control over VBM moving between secured areas of a facility as well as internal delivery associated with shipping and receiving processes. External transport security should ensure appropriate authorization and communication between facilities before, during and after external transport, which may involve the commercial transportation system. The recommendations of the United Nations Model Regulations for the Transport of Dangerous Goods (*23*), providing countries with a framework for the development of

CLUSTER	SPECIFIC REQUIREMENTS			IMPLEMENTATION
	UNSC Res. 1540	BTWC	IHR (2005)	
				national and international transport regulations include provisions addressing the security of dangerous goods, including infectious substances, during transport by all modes.
ZOONOTIC DISEASES			***JEE Technical Area 4: Zoonotic disease***<br>→ *Functional multisectoral, multidisciplinary mechanisms, policies, systems and practices are in place to minimize the transmission of zoonotic diseases from animals to human populations.*	
			Agreement by the animal health and public health sectors on a common **list of zoonotic diseases/pathogens of greatest national public health concern.**	
			**Zoonotic surveillance systems** in place for priority zoonotic diseases/pathogens of	

CLUSTER	SPECIFIC REQUIREMENTS			IMPLEMENTATION
	UNSC Res. 1540	BTWC	IHR (2005)	
			greatest public health concern.	
			**Collaboration, coordination and communication** between the animal health and public health sectors for **preparedness, detection, assessment and response to zoonotic diseases** (e.g. strategy or plan for responding to zoonotic events in the animal health and public health sectors, MoU between the sectors for the management of zoonotic events, mechanisms for establishing interagency response teams).	
FOOD SAFETY			***JEE Technical Area 5: Food safety***<br>→ *Timely detection and effective response of potential food-related events in collaboration with other sectors*	

CLUSTER	SPECIFIC REQUIREMENTS			IMPLEMENTATION
	UNSC Res. 1540	BTWC	IHR (2005)	
			responsible for food safety.	
			Existence of indicator-based disease surveillance or event-based disease **surveillance** and supporting laboratory analysis to detect and assign aetiology for foodborne diseases or origin of contamination event, and investigation of hazards in foods linked to cases, outbreaks or events.	
TRAINING AND EXERCISES			***JEE Technical Area 6*: (Biosafety and) Biosecurity**<br>*→ A whole-of-government multisectoral national (biosafety and) biosecurity system (for all sectors including human, animal and agriculture facilities) with dangerous*	

CLUSTER	SPECIFIC REQUIREMENTS			IMPLEMENTATION
	UNSC Res. 1540	BTWC	IHR (2005)	
			pathogens[26] identified, held, secured and monitored in a minimal number of facilities according to best practices; biological risk management training and educational outreach conducted to promote a shared culture of responsibility[27], reduce dual-use risks, mitigate biological proliferation and deliberate use threats, and ensure safe transfer of biological agents; and country- specific (biosafety and) biosecurity legislation, laboratory licensing and pathogen control measures in place as appropriate.	
			**JEE Technical Area 6**	

[26] "Dangerous pathogens and toxins – The informal Australia Group provides a List of human and animal pathogens and toxins for export control (http://www.australiagroup.net/en/human_animal_pathogens.html, accessed 23 November 2017)", *Ibidem*
[27] "Responsible life sciences research for global health security: a guidance document. WHO/HSE/GAR/BDP/2010.2. Geneva: World Health organization; 2010 (http://www.who.int/csr/resources/publications/HSE_GAR_BDP_2010_2/en/ (accessed 21 November 2017)", *Ibidem*

CLUSTER	SPECIFIC REQUIREMENTS			IMPLEMENTATION
	UNSC Res. 1540	BTWC	IHR (2005)	
			Country has in place academic training proportionate to the assessed risks, including institutions that train those who maintain or work with dangerous pathogens and toxins	
			JEE Technical Area 6<br><br>Country has training programmes in place at all facilities and staff trained proportionate to the assessed risks, including those that house or work with dangerous pathogens and toxins	
			JEE Technical Area 6<br><br>Review of training needs assessment is conducted periodically and refresher training on needs areas are conducted periodically	
			JEE Technical Area 6<br><br>Training on emergency response procedures are provided periodically	**WHO Laboratory biosecurity guidance, Chapter 7. Training**<br><br>Laboratory biosecurity training, complementary to laboratory biosafety

CLUSTER	SPECIFIC REQUIREMENTS			IMPLEMENTATION
	UNSC Res. 1540	BTWC	IHR (2005)	
				training and commensurate with the roles, responsibilities and authorities of staff, should be provided to all those working at a facility, including maintenance and cleaning personnel, and to external first-responders and responsible staff involved in ensuring the security of the laboratory facility.<br>The biorisk management plan should ensure that laboratory personnel and external partners (police, fire brigade, medical emergency personnel) participate actively in laboratory biosecurity drills and exercises, conducted at regular intervals, to revise emergency procedures and prepare personnel for emergencies.
BORDER AND EXPORT CONTROLS	***Para 3, subpara (c)(d)*** *→ Border controls and export and trans-shipment controls to prevent the proliferation of nuclear, chemical and biological weapons and their means of delivery including related materials*	***Article III*** → *Not to transfer, or in any way assist, encourage or induce anyone else to acquire or retain biological weapons.*	***JEE Technical Area 14: Linking public health and security authorities (Cluster - Response) →***<br>*Development and implementation of a MoU or other similar framework outlining roles, responsibilities and best practices for sharing relevant information between and among*	From the Annex XVII of the 1540 Committee's Report S/**2008**/493:<br>- WCO Framework of Standards to Secure and Facilitate Global Trade (the WCO SAFE Framework of Standards);<br>- International Standards for Phytosanitary Measures, under the International Plant Protection Convention, and similar measures for animal and human health

CLUSTER	SPECIFIC REQUIREMENTS			IMPLEMENTATION
	UNSC Res. 1540	BTWC	IHR (2005)	
			appropriate human and animal health, law enforcement and defence personnel, and validation of the MoU through periodic exercises and simulations.<br><br>***JEE Technical Area 6*: (Biosafety and) Biosecurity →**<br><br>*A whole-of-government multisectoral national (biosafety and) biosecurity system (for all sectors including human, animal and agriculture facilities) with dangerous pathogens[28] identified, held, secured and monitored in a minimal number of facilities according to best practices; biological risk management training and educational outreach*	From the Annex XVII of the 1540 Committee's Report S/**2011**/579:<br><br>- European Commission Taxation and Customs Union guidelines for export procedures and exit formalities

[28] "Dangerous pathogens and toxins – The informal Australia Group provides a List of human and animal pathogens and toxins for export control (http://www.australiagroup.net/en/human_animal_pathogens.html, accessed 23 November 2017)", *Ibidem*

CLUSTER	SPECIFIC REQUIREMENTS			IMPLEMENTATION
	**UNSC Res. 1540**	**BTWC**	**IHR (2005)**	
			conducted to promote a shared culture of responsibility[29]*, reduce dual-use risks, mitigate biological proliferation and deliberate use threats, and ensure safe transfer of biological agents; and country- specific (biosafety and) biosecurity legislation, laboratory licensing and pathogen control measures in place as appropriate.*	
	Border control to detect, deter, prevent and combat illicit trafficking		**JEE Technical Area 14**<br>Public Health and Security Authorities, (e.g. Law Enforcement, Border Control, Customs) are linked during a suspect or confirmed biological event, including through SOPs or agreements for coordination of a joint response to public health and other emergencies at	

[29] "Responsible life sciences research for global health security: a guidance document. WHO/HSE/GAR/BDP/2010.2. Geneva: World Health organization; 2010 (http://www.who.int/csr/resources/publications/HSE_GAR_BDP_2010_2/en/ (accessed 21 November 2017)", *Ibidem*

CLUSTER	SPECIFIC REQUIREMENTS			IMPLEMENTATION
	UNSC Res. 1540	BTWC	IHR (2005)	
			official locations, such as points of entry where both public health and security authorities have operational safety and health security responsibilities.	
	Law enforcement to detect, deter, **prevent** and combat illicit trafficking		Same as above	
	Export control legislation in place	The Sixth and Seventh Review Conferences called for appropriate measures, including effective national **export controls,** by all States Parties to implement this Article, in order to ensure that **direct and indirect transfers** relevant to the Convention, to any recipient whatsoever, are authorized only when the intended use is for purposes not prohibited under the Convention.		**Regulation (EU) 2021/821 of the European Parliament and of the Council setting up a Union regime for the control of exports, brokering, technical assistance, transit and transfer of dual-use items**<br><br>and<br><br>**Council Common Position 2008/944/CFSP of 8 December 2008 defining common rules governing control of exports of military technology and equipment**

CLUSTER	SPECIFIC REQUIREMENTS			IMPLEMENTATION
	UNSC Res. 1540	BTWC	IHR (2005)	
		**CBM Guide to Participating in the CBMs**<br><br>"Scope of Form E - Implementation is understood to include all measures that help to meet the aims and objectives of the Convention....Form E addresses: ...<br><br>2. Legislation and regulation relevant to Article III of the Convention (transfers), for example, legislation on export and import of micro-organisms and/or toxins pathogenic to humans, animals and plants in accordance with the Convention..."		
	Licensing provisions and Authority	Same as above		**Regulation (EU) 2021/821**<br><br>**LICENSING PROVISIONS**<br><br>***Article 3***<br><br>1. An authorisation shall be required for the export of dual-use items listed in Annex I.<br><br>2. Pursuant to Article 4, 5, 9 or 10, an authorisation may also be required for the

CLUSTER	SPECIFIC REQUIREMENTS			IMPLEMENTATION
	UNSC Res. 1540	BTWC	IHR (2005)	
				export to all or certain destinations of certain dual-use items not listed in Annex I.<br><br>***Article 4***<br><br>1. An authorisation shall be required for the export of dual-use items not listed in Annex I if the exporter has been informed by the competent authority that the items in question are or may be intended, in their entirety or in part:<br><br>(a) for use in connection with the development, production, handling, operation, maintenance, storage, detection, identification or dissemination of chemical, biological or nuclear weapons or other nuclear explosive devices or the development, production, maintenance or storage of missiles capable of delivering such weapons;<br><br>(b) for a military end-use if the purchasing country or country of destination is subject to an arms embargo; for the purposes of this point, 'military end-use' means:<br><br>(i) incorporation into military items listed in the military list of Member States;<br><br>(ii) use of production, test or analytical equipment and components therefor, for the development, production or maintenance of

CLUSTER	SPECIFIC REQUIREMENTS			IMPLEMENTATION
	UNSC Res. 1540	BTWC	IHR (2005)	
				military items listed in the military list of Member States; or<br><br>(iii) use of any unfinished products in a plant for the production of military items listed in the military list of Member States;<br><br>(c) for use as parts or components of military items listed in the national military list that have been exported from the territory of a Member State without authorisation or in violation of an authorisation prescribed by the national legislation of that Member State.<br><br>2. Where an exporter is aware that dual-use items which he proposes to export, not listed in Annex I, are intended, in their entirety or in part, for any of the uses referred to in paragraph 1 of this Article, the exporter shall notify the competent authority. That competent authority shall decide whether or not to make the export concerned subject to authorisation.<br><br>3. A Member State may adopt or maintain national legislation imposing an authorisation requirement on the export of dual-use items not listed in Annex I if the exporter has grounds for suspecting that those items are or may be intended, in their entirety or in part, for any of the uses referred to in paragraph 1 of this Article.

CLUSTER	SPECIFIC REQUIREMENTS			IMPLEMENTATION
	UNSC Res. 1540	BTWC	IHR (2005)	
				4. A Member State which imposes an authorisation requirement pursuant to paragraph 1, 2, or 3 shall immediately inform its customs authorities and other relevant national authorities and provide the other Member States and the Commission with relevant information on the authorisation requirement in question, in particular as regards the items and end-users concerned, unless it considers that it is not appropriate to do so in light of the nature of the transaction or the sensitivity of the information concerned.<br><br>5. Member States shall give due consideration to information received pursuant to paragraph 4 and shall inform their customs authorities and other relevant national authorities thereof.<br><br>6. In order to allow for an examination of all valid denials by the Member States, Article 16(1), (2) and (5) to (7) shall apply to cases concerning dual-use items not listed in Annex I.<br><br>7. All exchanges of information required pursuant to this Article shall take place in accordance with the legal requirements concerning the protection of personal information, commercially sensitive information or protected defence, foreign policy or national security information. Such

CLUSTER	SPECIFIC REQUIREMENTS			IMPLEMENTATION
	UNSC Res. 1540	BTWC	IHR (2005)	
				exchanges of information shall be made via secure electronic means, including through the system referred to in Article 23(6).<br><br>8. This Regulation is without prejudice to the right of Member States to take national measures under Article 10 of Regulation (EU) 2015/479.<br><br>***Article 6***<br><br>1. An authorisation shall be required for the provision of brokering services of dual-use items listed in Annex I if the broker has been informed by the competent authority that the items in question are or may be intended, in their entirety or in part, for any of the uses referred to in Article 4(1).<br><br>2. Where a broker proposes to provide brokering services of dual-use items listed in Annex I and is aware that those items are intended, in their entirety or in part, for any of the uses referred to in Article 4(1), the broker shall notify the competent authority. That competent authority shall decide whether or not to make such brokering services subject to authorisation.

CLUSTER	SPECIFIC REQUIREMENTS			IMPLEMENTATION
	UNSC Res. 1540	BTWC	IHR (2005)	
				3. A Member State may extend the application of paragraph 1 to non-listed dual-use items.<br><br>4. A Member State may adopt or maintain national legislation imposing an authorisation requirement on the provision of brokering services of dual-use items if the broker has grounds for suspecting that those items are or may be intended for any of the uses referred to in Article 4(1).<br><br>5. Article 9(2), (3) and (4) shall apply to the national measures referred to in paragraphs 3 and 4 of this Article.<br><br>***Article 8***<br><br>1. An authorisation shall be required for the provision of technical assistance related to dual-use items listed in Annex I if the provider of technical assistance has been informed by the competent authority that the items in question are or may be intended, in their entirety or in part, for any of the uses referred to in Article 4(1).<br><br>2. Where a provider of technical assistance proposes to provide technical assistance for dual-use items listed in Annex I and is aware that those items are intended, in their entirety

CLUSTER	SPECIFIC REQUIREMENTS			IMPLEMENTATION
	UNSC Res. 1540	BTWC	IHR (2005)	
				or in part, for any of the uses referred to in Article 4(1), the provider of technical assistance shall notify the competent authority. That competent authority shall decide whether or not to make such technical assistance subject to authorisation.<br><br>3. Paragraphs 1 and 2 shall not apply if the technical assistance:<br><br>(a) is provided within or into the territory of a country listed in Part 2 of Section A of Annex II, or towards a resident of a country listed in Part 2 of Section A of Annex II;<br><br>(b) takes the form of transferring information that is in the public domain or basic scientific research within the meaning of the General Technology Note or of the Nuclear Technology Note set out in Annex I;<br><br>(c) is provided by authorities or agencies of a Member State in the context of their official tasks;<br><br>(d) is provided for the armed forces of a Member State on the basis of the tasks assigned to them;<br><br>(e) is provided for a purpose which is cited in the exceptions for items of the Missile Technology Control Regime (MTCR technology) in Annex IV; or

CLUSTER	SPECIFIC REQUIREMENTS			IMPLEMENTATION
	UNSC Res. 1540	BTWC	IHR (2005)	
				(f) is the minimum necessary for the installation, operation, maintenance (checking) or repair of those items for which an export authorisation has been issued.<br><br>4. A Member State may extend the application of paragraph 1 to non-listed dual-use items.<br><br>5. A Member State may adopt or maintain national legislation imposing an authorisation requirement on the provision of technical assistance where a provider of technical assistance who proposes to provide technical assistance for dual-use items has grounds for suspecting that those items are or may be intended for any of the uses referred to in Article 4(1).<br><br>6. Article 9(2), (3) and (4) shall apply to the national measures referred to in paragraphs 4 and 5 of this Article.<br><br>***Article 9***<br><br>1. A Member State may prohibit or impose an authorisation requirement on the export of dual-use items not listed in Annex I for reasons of public security, including the prevention of acts of terrorism, or for human rights considerations.

CLUSTER	SPECIFIC REQUIREMENTS			IMPLEMENTATION
	UNSC Res. 1540	BTWC	IHR (2005)	
				2. Member States shall notify the Commission and the other Member States of any measures adopted pursuant to paragraph 1 without delay and indicate the precise reasons for the measures. If the measure is the establishment of a national control list, Member States shall also inform the Commission and the other Member States of the description of the controlled items.<br><br>3. Member States shall, without delay, notify the Commission and the other Member States of any amendment to measures adopted pursuant to paragraph 1, including any amendment to their national control lists.<br><br>4. The Commission shall publish the measures notified to it pursuant to paragraphs 2 and 3 in the C series of the *Official Journal of the European Union*. The Commission shall publish separately, without delay and in all the official languages of the Union, a compilation of national control lists in force in the Member States. The Commission shall, upon notification by a Member State of any amendment to its national control list, publish, without delay and in all the official languages of the Union, an update to the compilation of national control lists in force in the Member States.

CLUSTER	SPECIFIC REQUIREMENTS			IMPLEMENTATION
	UNSC Res. 1540	BTWC	IHR (2005)	
				Article 10<br><br>1. An authorisation shall be required for the export of dual-use items not listed in Annex I if another Member State imposes an authorisation requirement for the export of those items on the basis of a national control list of items adopted by that Member State pursuant to Article 9 and published by the Commission pursuant to Article 9(4), and if the exporter has been informed by the competent authority that the items in question are or may be intended, in their entirety or in part, for uses of concern with respect to public security, including the prevention of acts of terrorism, or to human rights considerations.<br><br>2. A Member State which refuses an authorisation required under paragraph 1 shall also inform the Commission and the other Member States of such decision.<br><br>3. A Member State which imposes an authorisation requirement pursuant to paragraph 1 of this Article on the export of a dual-use item not listed in Annex I, shall inform its customs authorities and other relevant national authorities about the authorisation requirement without delay and, where appropriate, provide the other Member States

CLUSTER	SPECIFIC REQUIREMENTS			IMPLEMENTATION
	UNSC Res. 1540	BTWC	IHR (2005)	
				and the Commission with the relevant information, in particular concerning the items and end-users concerned. The other Member States shall give due consideration to that information and shall inform their customs authorities and other relevant national authorities thereof.<br>***Article 11***<br>1. An authorisation shall be required for intra-Union transfers of dual-use items listed in Annex IV. Dual-use items listed in Part 2 of Annex IV shall not be covered by a general authorisation.<br>2. A Member State may impose an authorisation requirement for the transfer of other dual-use items from its territory to another Member State in cases where at the time of transfer:<br>(a) the operator or the competent authority knows that the final destination of the items concerned is outside the customs territory of the Union;<br>(b) the export of those items to that final destination is subject to an authorisation requirement pursuant to Article 3, 4, 5, 9 or 10 in the Member State from which the items are to be transferred, and such export directly

CLUSTER	SPECIFIC REQUIREMENTS			IMPLEMENTATION
	UNSC Res. 1540	BTWC	IHR (2005)	
				from its territory is not authorised by a general authorisation or a global authorisation; and<br><br>(c) no processing or working as defined in Article 60(2) of the Union Customs Code is to be performed on the items in the Member State to which they are to be transferred.<br><br>3. The transfer authorisation referred to in paragraphs 1 and 2 shall be applied for in the Member State from which the dual-use items are to be transferred.<br><br>4. In cases where the subsequent export of the dual-use items has already been accepted in the consultation procedures set out in Article 14 by the Member State from which the items are to be transferred, the transfer authorisation shall be issued to the operator immediately, unless the circumstances have substantially changed.<br><br>5. A Member State which adopts legislation imposing an authorisation requirement as referred to in paragraph 2 shall, without delay, inform the Commission and the other Member States of the measures it has taken. The Commission shall publish that information in the C series of the *Official Journal of the European Union*.

CLUSTER	SPECIFIC REQUIREMENTS			IMPLEMENTATION
	UNSC Res. 1540	BTWC	IHR (2005)	
				6. The application of measures taken pursuant to paragraphs 1 and 2 shall not involve the application of internal frontier controls within the customs territory of the Union, but solely controls which are performed as part of the normal control procedures applied in a non-discriminatory fashion throughout the customs territory of the Union.<br><br>7. The application of measures taken pursuant to paragraphs 1 and 2 shall not result in transfers from one Member State to another being subject to more restrictive conditions than those imposed for exports of the same items to third countries.<br><br>8. A Member State may, by national legislation, require that, for any intra-Union transfers from that Member State of items listed in Annex I, Category 5, Part 2, which are not listed in Annex IV, additional information concerning those items shall be provided to the competent authority of that Member State.<br><br>9. The relevant commercial documents relating to intra-Union transfers of dual-use items listed in Annex I shall indicate clearly that those items are subject to controls if exported from the customs territory of the Union. Such documents include, in particular, any sales

CLUSTER	SPECIFIC REQUIREMENTS			IMPLEMENTATION
	UNSC Res. 1540	BTWC	IHR (2005)	
				contract, order confirmation, invoice or dispatch note.<br><br>**AUTHORITY**<br>***Article 12***<br>1. The following types of authorisations for export may be issued or are established under this Regulation:<br>(a) individual export authorisations;<br>(b) global export authorisations;<br>(c) national general export authorisations;<br>(d) Union general export authorisations for exports of certain items to certain destinations under specific conditions and requirements for use as set out in Sections A to H of Annex II.<br>Authorisations issued or established under this Regulation shall be valid throughout the customs territory of the Union.<br>2. Individual and global export authorisations under this Regulation shall be granted by the competent authority of the Member State where the exporter is resident or established.<br>Without prejudice to point (3) of Article 2, where the exporter is not resident or established on the customs territory of the Union, individual export authorisations shall be

CLUSTER	SPECIFIC REQUIREMENTS			IMPLEMENTATION
	UNSC Res. 1540	BTWC	IHR (2005)	
				granted under this Regulation by the competent authority of the Member State where the dual-use items are located.<br><br>All individual and global export authorisations shall be issued, whenever possible, by electronic means on forms containing at least all the elements of and in the order provided for in the models set out in Section A of Annex III.
	Control of brokering	Same as above		**Regulation (EU) 2021/821**<br><br>***Article 2, point 7***<br><br>(7) 'brokering services' means:<br><br>(a) the negotiation or arrangement of transactions for the purchase, sale or supply of dual-use items from a third country to any other third country; or<br><br>(b) the selling or buying of dual-use items that are located in third countries for their transfer to another third country.<br><br>For the purposes of this Regulation, the sole provision of ancillary services is excluded from this definition. Ancillary services are transportation, financial services, insurance or re-insurance, or general advertising or promotion;

CLUSTER	SPECIFIC REQUIREMENTS			IMPLEMENTATION
	UNSC Res. 1540	BTWC	IHR (2005)	
				Article 6<br>1. An authorisation shall be required for the provision of brokering services of dual-use items listed in Annex I if the broker has been informed by the competent authority that the items in question are or may be intended, in their entirety or in part, for any of the uses referred to in Article 4(1).<br>2. Where a broker proposes to provide brokering services of dual-use items listed in Annex I and is aware that those items are intended, in their entirety or in part, for any of the uses referred to in Article 4(1), the broker shall notify the competent authority. That competent authority shall decide whether or not to make such brokering services subject to authorisation.<br>3. A Member State may extend the application of paragraph 1 to non-listed dual-use items.<br>4. A Member State may adopt or maintain national legislation imposing an authorisation requirement on the provision of brokering services of dual-use items if the broker has grounds for suspecting that those items are or

CLUSTER	SPECIFIC REQUIREMENTS			IMPLEMENTATION
	UNSC Res. 1540	BTWC	IHR (2005)	
				may be intended for any of the uses referred to in Article 4(1).<br><br>5. Article 9(2), (3) and (4) shall apply to the national measures referred to in paragraphs 3 and 4 of this Article.<br><br>***Article 13***<br><br>1. Authorisations for the provision of brokering services and technical assistance under this Regulation shall be granted by the competent authority of the Member State where the broker or the provider of technical assistance is resident or established. Where the broker or the provider of technical assistance is not resident or established on the customs territory of the Union, authorisations for the provision of brokering services and technical assistance under this Regulation shall be granted by the competent authority of the Member State from where the brokering services or technical assistance will be provided.<br><br>2. Authorisations for the provision of brokering services shall be granted for a set quantity of specific items and shall clearly identify the location of the items in the

CLUSTER	SPECIFIC REQUIREMENTS			IMPLEMENTATION
	UNSC Res. 1540	BTWC	IHR (2005)	
				originating third country, the end-user and the exact location of the end-user.<br><br>Authorisations for technical assistance shall clearly identify the end-user and the exact location of the end-user.<br><br>The authorisations shall be valid throughout the customs territory of the Union.<br><br>3. Brokers and providers of technical assistance shall supply the competent authority with all relevant information required for their application for authorisation under this Regulation, in particular details of the location of the dual-use items, a clear description of the items and the quantity involved, third parties involved in the transaction, the country of destination, the end-user in that country and its exact location.<br><br>4. The competent authorities of the Member States shall process requests for authorisations for the provision of brokering services and technical assistance within a period of time to be determined by national law or practice.<br><br>5. All authorisations for the provision of brokering services and technical assistance shall be issued, whenever possible, by electronic means on forms containing at least all the elements of and in the order provided

CLUSTER	SPECIFIC REQUIREMENTS			IMPLEMENTATION
	UNSC Res. 1540	BTWC	IHR (2005)	
				for in the models set out in Section B of Annex III.<br><br>***Article 16***<br><br>1. The competent authority acting in accordance with this Regulation, may refuse to grant an export authorisation and may annul, suspend, modify or revoke an export authorisation which it has already granted. Where the competent authority refuses, annuls, suspends, substantially limits or revokes an export authorisation or when it has determined that the intended export is not to be authorised, it shall notify the competent authorities of the other Member States and the Commission thereof and share the relevant information with them. In case the competent authority of a Member State has suspended an export authorisation, the final assessment shall be communicated to the competent authorities of the other Member States and the Commission at the end of the period of suspension.<br><br>2. The competent authorities of the Member States shall review denials of authorisations notified under paragraph 1 within three years of their notification and revoke them, amend them or renew them. The

CLUSTER	SPECIFIC REQUIREMENTS			IMPLEMENTATION
	UNSC Res. 1540	BTWC	IHR (2005)	
				competent authorities of the Member States shall notify the results of the review to the competent authorities of the other Member States and the Commission as soon as possible. Denials which are not revoked shall remain valid and shall be reviewed every three years. At the third review, the Member State concerned shall be required to explain the reasoning for maintaining such denial.<br><br>3. The competent authority shall notify the competent authorities of the other Member States and the Commission of their decisions to prohibit a transit of dual-use items taken under Article 7 without delay. These notifications shall contain all relevant information including the classification of the item, its technical parameters, the country of destination and the end-user.<br><br>4. Paragraphs 1 and 2 of this Article shall also apply to authorisations for the provision of brokering services and technical assistance referred to in Article 13.<br><br>***Article 28***<br><br>In order to ensure that this Regulation is properly applied, each Member State shall take

CLUSTER	SPECIFIC REQUIREMENTS			IMPLEMENTATION
	UNSC Res. 1540	BTWC	IHR (2005)	
				all necessary measures to permit its competent authorities:<br>(a) to gather information on any order or transaction involving dual-use items;<br>(b) to establish whether the export control measures are being properly applied, which may include in particular the power to enter the premises of persons with an interest in an export transaction or brokers involved in the provision of brokering services under circumstances set out in Article 6, or providers of technical assistance under the circumstances set out in Article 8.
	Intangible technology transfers	Same as above	JEE Technical Area 6<br>There is a mechanism for biosecurity oversight of dual-use research and responsible code of conduct for scientists in place.	**WHO Laboratory biosecurity guidance, Chapter 5. Countering biorisk, 5.3 Legitimate research, codes of conduct and codes of practice**<br>The code of conduct should involve evaluation of the purpose of the work, consideration for its impact the publication of research results, and enumerate considerations and conditions for or against the publication of results that may have dual-use implications<br><br>**Regulation (EU) 2021/821**

CLUSTER	SPECIFIC REQUIREMENTS			IMPLEMENTATION
	UNSC Res. 1540	BTWC	IHR (2005)	
				**Article 2, point 2, item (d)**<br>'export' means:<br>...<br>(d) transmission of software or technology by electronic media, including by fax, telephone, electronic mail or any other electronic means to a destination outside the customs territory of the Union; it includes making available in an electronic form such software and technology to natural or legal persons or to partnerships outside the customs territory of the Union; it also includes the oral transmission of technology when the technology is described over a voice transmission medium;<br><br>**Article 3, para 1**<br>1. An authorisation shall be required for the export of the dual-use items listed in Annex I.
	Inclusion of means of delivery	Same as above		**Regulation (EU) 2021/821**<br>**Annex I**<br>Part III - Category 1 Special materials and related equipment<br>Part IX - Category 7 Navigation and avionics<br>Part X - Category 8 Marine

CLUSTER	SPECIFIC REQUIREMENTS			IMPLEMENTATION
	**UNSC Res. 1540**	**BTWC**	**IHR (2005)**	
				Part XI - Category 9 Aerospace and propulsion
	End-user controls	Same as above		**Regulation (EU) 2021/821**<br>**Article 12, para 4**<br>4. Exporters shall supply the competent authority with all relevant information required for their applications for individual and global export authorisations so as to provide complete information in particular about the end-user, the country of destination and the end-use of the item exported.<br>**Article 13, para 2 and 3**<br>2. Authorisations for the provision of brokering services shall be granted for a set quantity of specific items and shall clearly identify the location of the items in the originating third country, the end-user and the exact location of the end-user.<br>Authorisations for technical assistance shall clearly identify the end-user and the exact location of the end-user.<br>The authorisations shall be valid throughout the customs territory of the Union.

CLUSTER	SPECIFIC REQUIREMENTS			IMPLEMENTATION
	UNSC Res. 1540	BTWC	IHR (2005)	
				3. Brokers and providers of technical assistance shall supply the competent authority with all relevant information required for their application for authorisation under this Regulation, in particular details of the location of the dual-use items, a clear description of the items and the quantity involved, third parties involved in the transaction, the country of destination, the end-user in that country and its exact location.
	Catch all clause	Same as above		**Regulation (EU) 2021/821**<br><br>***Article 4 , para 1-3***<br><br>1. An authorisation shall be required for the export of dual-use items not listed in Annex I if the exporter has been informed by the competent authority that the items in question are or may be intended, in their entirety or in part:<br><br>(a) for use in connection with the development, production, handling, operation, maintenance, storage, detection, identification or dissemination of chemical, biological or nuclear weapons or other nuclear explosive devices or the development, production, maintenance or storage of missiles capable of delivering such weapons;

CLUSTER	SPECIFIC REQUIREMENTS			IMPLEMENTATION
	UNSC Res. 1540	BTWC	IHR (2005)	
				(b) for a military end-use if the purchasing country or country of destination is subject to an arms embargo; for the purposes of this point, 'military end-use' means:<br><br>(i) incorporation into military items listed in the military list of Member States;<br><br>(ii) use of production, test or analytical equipment and components therefor, for the development, production or maintenance of military items listed in the military list of Member States; or<br><br>(iii) use of any unfinished products in a plant for the production of military items listed in the military list of Member States;<br><br>(c) for use as parts or components of military items listed in the national military list that have been exported from the territory of a Member State without authorisation or in violation of an authorisation prescribed by the national legislation of that Member State.<br><br>2. Where an exporter is aware that dual-use items which he proposes to export, not listed in Annex I, are intended, in their entirety or in part, for any of the uses referred to in paragraph 1 of this Article, the exporter shall notify the competent authority. That competent authority shall decide whether or

CLUSTER	SPECIFIC REQUIREMENTS			IMPLEMENTATION
	UNSC Res. 1540	BTWC	IHR (2005)	
				not to make the export concerned subject to authorisation.<br><br>3. A Member State may adopt or maintain national legislation imposing an authorisation requirement on the export of dual-use items not listed in Annex I if the exporter has grounds for suspecting that those items are or may be intended, in their entirety or in part, for any of the uses referred to in paragraph 1 of this Article.<br><br>...<br><br>***Article 9, para 1***<br><br>1. A Member State may prohibit or impose an authorisation requirement on the export of dual-use items not listed in Annex I for reasons of public security, including the prevention of acts of terrorism, or for human rights consideration<br><br>....<br><br>***Article 6, para 1 and 2***<br><br>1. An authorisation shall be required for the provision of brokering services of dual-use items listed in Annex I if the broker has been informed by the competent authority that the items in question are or may be intended, in

CLUSTER	SPECIFIC REQUIREMENTS			IMPLEMENTATION
	UNSC Res. 1540	BTWC	IHR (2005)	
				their entirety or in part, for any of the uses referred to in Article 4(1).<br><br>2. Where a broker proposes to provide brokering services of dual-use items listed in Annex I and is aware that those items are intended, in their entirety or in part, for any of the uses referred to in Article 4(1), the broker shall notify the competent authority. That competent authority shall decide whether or not to make such brokering services subject to authorisation.<br><br>...
	Transit control	Same as above		**Regulation (EU) 2021/821**<br><br>***Article 7***<br><br>1. The transit of non-Union dual-use items listed in Annex I may be prohibited at any time by the competent authority of the Member State where the items are situated if the items are or may be intended, in their entirety or in part, for any of the uses referred to in Article 4(1).<br><br>2. Before deciding whether or not to prohibit a transit the competent authority may, in individual cases, impose an authorisation requirement for the specific transit of dual-use items listed in Annex I if the items are or may be intended, in their entirety or in part, for any

CLUSTER	SPECIFIC REQUIREMENTS			IMPLEMENTATION
	UNSC Res. 1540	BTWC	IHR (2005)	
				of the uses referred to in Article 4(1). If the transit takes place through the territory of multiple Member States, the competent authority of each affected Member State shall be able to prohibit such transit through its territory.<br><br>The competent authority may impose the authorisation requirement on the natural or legal person or the partnership that holds the contract with the consignee in the third country and has the power to determine the sending of the items passing through the customs territory of the Union.<br><br>If the natural or legal person or the partnership is not resident or established in the customs territory of the Union, the competent authority may impose the authorisation requirement on:<br><br>(a) the declarant within the meaning of Article 5(15) of the Union Customs Code;<br><br>(b) the carrier within the meaning of Article 5(40) of the Union Customs Code; or<br><br>(c) the natural person carrying the dual-use items in transit where those dual-use items are contained in the personal baggage of that person.

CLUSTER	SPECIFIC REQUIREMENTS			IMPLEMENTATION
	UNSC Res. 1540	BTWC	IHR (2005)	
				3. A Member State may extend the application of paragraph 1 to non-listed dual-use items.<br><br>4. Article 9(2), (3) and (4) shall apply to the national measures referred to in paragraph 3 of this Article.<br><br>***Article 15***<br><br>1. In deciding whether or not to grant an authorisation or to prohibit a transit under this Regulation, the Member States shall take into account all relevant considerations, including:<br><br>(a) Union and Member States' international obligations and commitments, in particular the obligations and commitments they have each accepted as members of the relevant international non-proliferation regimes and export control arrangements, or by ratification of relevant international treaties;<br><br>(b) their obligations under sanctions imposed by a decision or a common position adopted by the Council or by a decision of the OSCE or by a binding resolution of the Security Council of the United Nations;

CLUSTER	SPECIFIC REQUIREMENTS			IMPLEMENTATION
	UNSC Res. 1540	BTWC	IHR (2005)	
				(c) considerations of national foreign and security policy, including those covered by Common Position 2008/944/CFSP;<br><br>(d) considerations about intended end-use and the risk of diversion.<br><br>2. In addition to the criteria set out in paragraph 1, when assessing an application for a global export authorisation, Member States shall take into consideration the implementation of an ICP by the exporter.
	Trans-shipment control	Same as above		Article 7 of the **Regulation (EU) 2021/821** would be applicable, as transit is defined in Article 2 in the following manner:<br><br>(11) 'transit' means a transport of non-Union dual-use items entering and passing through the customs territory of the Union with a destination outside the customs territory of the Union where those items:<br><br>(a) are placed under an external transit procedure according to Article 226 of the Union Customs Code and only pass through the customs territory of the Union;<br><br>(b) are trans-shipped within, or directly re-exported from, a free zone;

CLUSTER	SPECIFIC REQUIREMENTS			IMPLEMENTATION
	UNSC Res. 1540	BTWC	IHR (2005)	
				(c) are in temporary storage and are directly re-exported from a temporary storage facility; or<br><br>(d) were brought into the customs territory of the Union on the same vessel or aircraft that will take them out of that territory without unloading;...
	Re-export control	Same as above		**Regulation (EU) 2021/821**<br><br>**Article 2, point 2, item (b)**<br><br>'(2) 'export' means:<br><br>...<br><br>(b) a re-export within the meaning of Article 270 of the Union Customs Code; a re-export also occurs if, during a transit through the customs territory of the Union according to point (11) of this Article, an exit summary declaration has to be lodged because the final destination of the items has been changed;<br><br>...<br><br>**Article 3, para 1**<br><br>1. An authorisation shall be required for the export of the dual-use items listed in Annex I.

CLUSTER	SPECIFIC REQUIREMENTS			IMPLEMENTATION
	UNSC Res. 1540	BTWC	IHR (2005)	
	Control over financing of exports/transhipments that would contribute to proliferation			From the Annex XVII of the 1540 Committee's Report S/**2011**/579:<br>- Financial Action Task Force (FATF) Guidance on Counter Proliferation Financing - The Implementation of Financial Provisions of United Nations Security Council Resolutions to Counter the Proliferation of Weapons of Mass Destruction<br>- FATF published its June 2008 Proliferation Financing Report,10 a typology study on the methods and techniques used to finance proliferation, including specific case examples and measures being taken by countries to combat this activity.
	Control over services (e.g. transport) related to exports/ transhipments that would contribute to proliferation			
	Criminal or civil penalties for violations of such export control laws and regulations			**Regulation (EU) 2021/821**<br>***Article 25***<br>1. Each Member State shall take appropriate measures to ensure the proper enforcement of this Regulation. In particular, it shall lay down

CLUSTER	SPECIFIC REQUIREMENTS			IMPLEMENTATION
	UNSC Res. 1540	BTWC	IHR (2005)	
				the penalties applicable to infringements of the provisions of this Regulation or of those adopted for its implementation. Those penalties shall be effective, proportionate and dissuasive.
RAISING AWARENESS	***Para 7* and *8, subpara (d)*** *→ Assistance, work with and inform to Industry and Public, and other Information*[30]	***Article IV*** *→ To take any national measures necessary to implement the provisions of the BTWC domestically*		In 2016 Germany submitted to the 1540 Committee a paper on best practices with regard to Germany's work with industry in the fields of export control (Note 376/29016[31] available on the 1540 Committee's website under "Experiences Shared, Lessons Learned, and Effective Practices"). Similar document ('Export Controls: The Importance of Engaging Industry') was submitted 2 years earlier together with Australia[32].
	Action taken to work with and inform industry	**Article IV** The Seventh Review Conference noted the value of national implementation measures, as appropriate, in accordance with the constitutional process of		

[30] Actions with regard to offers and request for assistance, which states and relevant international organization are called to undertake under operative paragraph 7 as well as appointment of the Point of Contact are not considered relevant in the context of this Project.
[31] https://www.un.org/en/sc/1540/documents/Germany2016.pdf
[32] https://www.un.org/en/sc/1540/documents/Australia-Germany%20Letter%20re%20effective%20practices%202014.pdf

CLUSTER	SPECIFIC REQUIREMENTS			IMPLEMENTATION
	**UNSC Res. 1540**	**BTWC**	**IHR (2005)**	
		each State Party, to... encourage the promotion of a culture of responsibility amongst relevant national professionals and the voluntary development, adoption and promulgation of codes of conduct.<br>The Sixth Review Conference recognised the importance of codes of conduct and self-regulatory mechanisms in raising awareness, and called upon States Parties to support and encourage their development, promulgation and adoption.		
	Action taken to work with and inform the public			
DIVERSION TO PEACEFUL PURPOSES		***Article II*** → *To destroy or divert to peaceful purposes all agents, toxins, weapons, equipment and means of delivery specified in Article I*		

CLUSTER	SPECIFIC REQUIREMENTS			IMPLEMENTATION
	UNSC Res. 1540	BTWC	IHR (2005)	
		prior to joining the Convention.		
		The Sixth and Seventh Review Conferences emphasised that states must take all necessary safety and security provisions to protect populations and the environment when carrying out such destruction and/or diversion. The Seventh Review Conference emphasised that these measures should protect human populations and the environment, including animals and plants.		

Table S2: Prohibitions & Penalties

<table>
<tr><th rowspan="2">CLUSTER</th><th colspan="3">SPECIFIC REQUIREMENTS</th><th rowspan="2">IMPLEMENTATION</th></tr>
<tr><th>UNSC Res. 1540</th><th>BTWC</th><th>IHR (2005)</th></tr>
<tr><td>PROHIBITIONS</td><td>Para 2 → National legislation which prohibits persons or entities to engage in one of the following activities and its enforcement</td><td>Article I → Never under any circumstances to acquire or retain biological weapons, equipment and means of delivery

Article III → Not to transfer, or in any way assist, encourage or induce anyone else to acquire or retain biological weapons.

The Fourth Review Conference noted that States Parties should also consider ways and means to ensure that individuals or subnational groups are effectively prevented from acquiring, through transfers, biological agents and toxins for other than peaceful purposes.

Article IV → To take any national measures necessary to implement the provisions of the BTWC domestically</td><td></td><td></td></tr>
<tr><td></td><td>manufacture</td><td rowspan="6">Article I

Use

The Sixth and Seventh Review Conferences reaffirmed the undertaking in Article I never in any circumstances to develop, produce, stockpile or otherwise acquire or retain weapons, equipment, or means of delivery designed to use such agents or</td><td></td><td></td></tr>
<tr><td></td><td>acquire</td><td></td><td></td></tr>
<tr><td></td><td>possess</td><td></td><td></td></tr>
<tr><td></td><td>develop</td><td></td><td></td></tr>
<tr><td></td><td>transport</td><td></td><td></td></tr>
<tr><td></td><td>transfer</td><td></td><td></td></tr>
</table>

CLUSTER	SPECIFIC REQUIREMENTS			IMPLEMENTATION
	UNSC Res. 1540	BTWC	IHR (2005)	
	use biological weapons and their means of delivery	toxins for hostile purposes or in armed conflict in order to exclude completely and forever the possibility of their use.<br><br>**Article III**<br>Transfer<br>Participate<br>Assist<br><br>**Article IV**<br>Produce<br>Acquire<br>retain, stockpile<br>Develop<br>The Seventh Review Conference noted the value of national implementation measures, as appropriate, in accordance with the constitutional process of each State Party, to... prevent anyone from developing, producing, stockpiling, or otherwise acquiring or retaining, transporting or transferring and using under any circumstances, biological agents and toxins,		
	participate as an accomplice in abovementioned activities			
	assist in abovementioned activities			

CLUSTER	SPECIFIC REQUIREMENTS			IMPLEMENTATION
	UNSC Res. 1540	BTWC	IHR (2005)	
		equipment, or their means of delivery for non-peaceful purposes.<br><br>**CBM Guide to Participating in the CBMs**<br><br>"Scope of Form E - Implementation is understood to include all measures that help to meet the aims and objectives of the Convention....Form E addresses:<br><br>Legislation and regulation relevant to Article I of the Convention (development, production, stockpiling, acquisition or retention of microbial or other biological agents, or toxins, weapons, equipment and means of delivery), for example, legislation transposing BWC in national law **or penal legislation...**"		
	attempt to engage in abovementioned activities			
	finance abovementioned activities			

Table S3: Detection

CLUSTER	SPECIFIC REQUIREMENTS			IMPLEMENTATION
	**UNSC Res. 1540**	**BTWC**	**IHR (2005)**	
DETECTION AT:<br>- FACILITY<br>- BORDER<br>- OTHER	***Parts of the Para 3, subpara (c)***	***Article IV***<br><br>*The Sixth and Seventh Review Conferences reaffirmed the commitment of States Parties to take the necessary national measures to strengthen methods and capacities for* ***surveillance and detection*** *of outbreaks of disease at the national, regional and international levels.*	***IHR Article 5, para 1*** → *"Each State Party shall develop, strengthen and maintain as soon as possible .... the capacity to detect, assess, notify and report events in accordance with these Regulations as specified in Annex 1..."*<br><br>***JEE Technical area 8: National laboratory system***[33]<br>→ *Surveillance with a national laboratory system, including all relevant sectors*[34]*, particularly human and animal health, and effective modern3 point-of-care and laboratory-based diagnostics.*<br><br>***JEE Technical area 9: Surveillance***[35]<br>→ *A functioning public health surveillance system capable of identifying potential events of concern for public health and health security*[36]	

[33] The National Laboratory System is a collaborative community of clinical laboratories, public health laboratories, and many individual partners who initiate tests and/or use test results (https://www.ncbi.nlm.nih.gov/pmc/ articles/PMC2846799/pdf/phr125s20018.pdf, accessed 24 November 2017). The same applies to the National Veterinary Laboratory System.

[34] Relevant sectors include private and public sectors, such as: all levels of the health care system (national, subnational and community/primary public health); NGOs; divisions/activities of other sectors which affect public health, such as ministries of agriculture (quarantine and movement control authority, import/export regulations, disease diagnosis and control financing, zoonosis, veterinary laboratory etc.), transport (transport policy, civil aviation, ports and maritime transport), trade and/or industry (food safety and quality control), foreign trade (consumer protection, control of compulsory standard enforcement), communication, defense, treasury or finance (customs), environment, interior, health, tourism; health, tourism; the home office; media; and regulatory bodies.

[35] Surveillance, means the systematic ongoing collection, collation and analysis of data for public health purposes and the timely dissemination of public health information for assessment and public health response as necessary.

[36] Each country has to de ne a "potential risk to public health", perform risk mapping and identify priority diseases.

CLUSTER	SPECIFIC REQUIREMENTS			IMPLEMENTATION
	UNSC Res. 1540	BTWC	IHR (2005)	
			JEE Technical Area 14: Linking public health and security authorities (Cluster - Response) → *Development and implementation of a MoU or other similar framework outlining roles, responsibilities and best practices for sharing relevant information between and among appropriate human and animal health, law enforcement and defence personnel, and validation of the MoU through periodic exercises and simulations.*	
		**Article IV**<br><br>The Sixth and Seventh Review Conferences reaffirmed the commitment of States Parties to take the necessary national measures to strengthen methods and capacities for **surveillance and detection** of outbreaks of disease at the national, regional and international levels.	**JEE Technical area 8**<br>Existence of a nationwide laboratory system able to reliably conduct at least five of the 10 core tests[37] on appropriately identified and collected outbreak specimens transported safely and securely to accredited laboratories from at least 80% of intermediate levels/districts in the country	

[37] 10 core tests: A list in each country includes six testing methods selected according to the IHR's immediately notifiable list and the WHO top 10 causes of death in low-income countries: polymerase chain reaction testing for influenza virus; virus culture for poliovirus; serology for HIV; microscopy for *Mycobacterium tuberculosis*; rapid diagnostic testing for Plasmodium spp.; and bacterial culture for *Salmonella enteritidis* serotype *typhi.* These six methods are critical to the detection of epidemic-prone emerging diseases. Competency in these methods is indicated by successful testing for the specific pathogens listed. The remaining four tests should be selected by the country on the basis of major national public health concerns.

CLUSTER	SPECIFIC REQUIREMENTS			IMPLEMENTATION
	**UNSC Res. 1540**	**BTWC**	**IHR (2005)**	
		Same as above	**JEE Technical area 8**<br>Existence of national quality laboratory standards and system for licencing laboratories.	
		Same as above	**JEE Technical area 9**<br>Surveillance for at least three core syndromes[38] indicative of potential public health emergencies conducted according to international standards.	
		Same as above	**JEE Technical area 9**<br>Regular analysis and reporting of surveillance data.	
	Border control to detect, deter, prevent and combat illicit trafficking	Same as above	**JEE Technical Area 14**<br>Public Health and Security Authorities, (e.g. Law Enforcement, Border Control, Customs) are linked during a suspect or confirmed biological event, including through SOPs or agreements for coordination of a joint response to public health and other emergencies at official locations, such as points of entry where both public health and security authorities have operational safety and health security responsibilities.	
	Law enforcement to detect, deter, prevent and	Same as above	**JEE Technical Area 14**<br>Public Health and Security Authorities, (e.g. Law Enforcement, Border Control, Customs) are linked during a	

[38] Internationally recognized standards for syndromic surveillance are available for the following five syndromes: (i) severe acute respiratory syndrome, (ii) acute flaccid paralysis, (iii) acute haemorrhagic fever, (iv) acute watery diarrhoea with dehydration, and (v) acute jaundice syndrome. Three core syndromes are chosen depending on national disease control priorities. The surveillance system should include epidemiological data and laboratory findings, which should be analysed by trained epidemiologists.

CLUSTER	SPECIFIC REQUIREMENTS			IMPLEMENTATION
	UNSC Res. 1540	BTWC	IHR (2005)	
	combat illicit trafficking		suspect or confirmed biological event, including through SOPs or agreements for coordination of a joint response to public health and other emergencies at official locations, such as points of entry where both public health and security authorities have operational safety and health security responsibilities.	

Table S4: Response

CLUSTER	SPECIFIC REQUIREMENTS			IMPLEMENTATION
	**UNSC Res. 1540**	**BTWC**	**IHR (2005)**	
EMERGENCY PREPAREDNESS			***IHR Article 13 (1)***<br>*"Each State arty shall develop, strengthen and maintain as soon as possible ...the capacity to respond promptly and effectively to public health risks and public health emergencies of international concern as set out in Annex 1."*<br><br>***JEE Technical area 12: Emergency preparedness***<br>*→ Multisectoral actors at national and subnational (local and intermediate) levels have a common understanding of the priority risks and ready for timely, effective and efficient emergency response operations for outbreaks and other emergencies.*<br><br>***JEE Technical Area 6: (Biosafety and) Biosecurity →***<br>*A whole-of-government multisectoral national (biosafety and) biosecurity system (for all sectors including human, animal and agriculture facilities) with dangerous pathogens*[39] *identified, held, secured and monitored in a minimal number of facilities according to best practices; biological risk management training and educational outreach conducted to promote a shared culture of responsibility*[40]*, reduce dual-use risks, mitigate biological proliferation and deliberate use threats, and ensure safe transfer*	

[39] "Dangerous pathogens and toxins – The informal Australia Group provides a List of human and animal pathogens and toxins for export control (http://www.australiagroup.net/en/human_animal_pathogens.html, accessed 23 November 2017)", *Ibidem*
[40] "Responsible life sciences research for global health security: a guidance document. WHO/HSE/GAR/BDP/2010.2. Geneva: World Health organization; 2010 (http://www.who.int/csr/resources/publications/HSE_GAR_BDP_2010_2/en/ (accessed 21 November 2017)", *Ibidem*

CLUSTER	SPECIFIC REQUIREMENTS			IMPLEMENTATION
	UNSC Res. 1540	BTWC	IHR (2005)	
			of biological agents; and country- specific (biosafety and) biosecurity legislation, laboratory licensing and pathogen control measures in place as appropriate.	
			**JEE Technical area 12**<br>Existence of national strategic multihazard emergency risk assessments and resource mapping	
			**JEE Technical area 12**<br>Existence of multihazard emergency response plans	
			**JEE Technical area 12**<br>Evidence, from exercises, after-action and other reviews of effective and efficient multisectoral emergency response operations for outbreaks and other public health emergencies.	
			**JEE Technical Area 6**<br>Site-specific biosecurity management programmes and supporting documents (manuals, SOPs, job aides, records) are available and include biosecurity, incident response and emergency plans.	
EMERGENCY RESPONSE OPERATIONS			***IHR Article 13 (1)***<br>*"Each State arty shall develop, strengthen and maintain as soon as possible ...the capacity to respond promptly and effectively to public health risks and public health emergencies of international concern as set out in Annex 1."*	

CLUSTER	SPECIFIC REQUIREMENTS			IMPLEMENTATION
	UNSC Res. 1540	BTWC	IHR (2005)	
			JEE Technical area 13: Emergency response operations<br>*→ Countries will have a coordination mechanism, incident management systems, exercise management programmes and public health emergency operation centre (EOC) functioning according to minimum common standards; maintaining trained, functioning, multisectoral rapid response teams, and trained EOC staff capable of activating a coordinated emergency response within 120 minutes of the identification of an emergency.*	
			**JEE Technical area 13**<br>Establishment of an emergency response coordination mechanism or incident management system.	
			**JEE Technical area 13**<br>Development of national health EOC plans and procedures.	
			**JEE Technical area 13**<br>Emergency response systems and decision-making have been tested and operating efficiently and effectively.	
RISK COMMUNICATION			***JEE Technical area 16: Risk communication***<br>*→States Parties use multilevel, multisectoral and multifaceted risk communication capacity for public health emergencies. Real-time exchange of information, advice and opinions during unusual and unexpected events and emergencies so that informed decisions to mitigate the effects of threats, and protective and preventative action can be made. This includes a*	

CLUSTER	SPECIFIC REQUIREMENTS			IMPLEMENTATION
	**UNSC Res. 1540**	**BTWC**	**IHR (2005)**	
			mix of communication and engagement strategies, such as media and social media communications, mass awareness campaigns, health promotion, social mobilization, stakeholder engagement and community engagement.	
			**JEE Technical area 16**<br>Formal government risk communications plans, arrangements and systems are in place.	
			**JEE Technical area 16**<br>Risk communication coordination platform and mechanisms for internal and partner communication are in place.	
			**JEE Technical area 16**<br>Existence of a system to gather information on perceptions, risky behaviours and misinformation to analyse public concerns and fears.	
POINTS OF ENTRY			***JEE Technical area 17: Points of entry***<br>*→ Timely detection of and effective response to any potential hazards that occur at points of entry.*<br><br>***JEE Technical Area 14: Linking public health and security authorities (Cluster - Response) →***<br>*Development and implementation of a MoU or other similar framework outlining roles, responsibilities and best practices for sharing relevant information between and among appropriate human and animal health, law enforcement and defence*	

CLUSTER	SPECIFIC REQUIREMENTS			IMPLEMENTATION
	UNSC Res. 1540	BTWC	IHR (2005)	
			personnel, and validation of the MoU through periodic exercises and simulations.	
			**JEE Technical area 17**<br>Public health emergency contingency plan for designated points of entry.	
			**JEE Technical area 17**<br>Core capacities prescribed in the IHR Annex 1B "1. At all times" are developed and functioning in an all-hazard and multisectoral approach.	
			**JEE Technical area 14**<br>There are SOPs or agreements in place for coordination of a joint response to public health and other emergencies at of cial locations, such as points of entry<br><br>where both public health and security authorities have operational safety and health security responsibilities.	
LAW ENFORCEMENT RESPONSE	***Parts of para 3, subpara (c)***		***JEE Technical area 14: Linking public health and security authorities***<br>→ *Country conducts a rapid, multisectoral response for any event of suspected or confirmed deliberate origin, including the capacity to link public health and law enforcement, and to provide timely international assistance.*	

CLUSTER	SPECIFIC REQUIREMENTS			IMPLEMENTATION
	UNSC Res. 1540	BTWC	IHR (2005)	
			**JEE Technical area 14**<br>Developed and implemented model systems to conduct and support joint epidemiological and criminal investigations to identify and respond to suspected biological incidents of deliberate origin (e.g. SOPs or emergency response plans that would include security authorities, Informational reports that are regularly shared with security authorities, etc.)	
	Border control to detect, deter, prevent and combat illicit trafficking		**JEE Technical Area 14**<br>Public Health and Security Authorities, (e.g. Law Enforcement, Border Control, Customs) are linked during a suspect or confirmed biological event, including through SOPs or agreements for coordination of a joint response to public health and other emergencies at official locations, such as points of entry where both public health and security authorities have operational safety and health security responsibilities.	
	Law enforcement to detect, deter, prevent and combat illicit trafficking		**JEE Technical Area 14**<br>Public Health and Security Authorities, (e.g. Law Enforcement, Border Control, Customs) are linked during a suspect or confirmed biological event, including through SOPs or agreements for coordination of a joint response to public health and other emergencies at official locations, such as points of entry where both public health and security authorities have operational safety and health security responsibilities.	

Table S5: International cooperation, participation in related legally binding instruments, mechanisms or IOS

CLUSTER	SPECIFIC REQUIREMENTS			IMPLEMENTATION
	**UNSC Res. 1540**	**BTWC**	**IHR (2005)**	
MEDICAL COUNTERMEASURES AND PERSONNEL DEPLOYMENT			***JEE Technical area 15: Medical countermeasures and personnel deployment***<br>*→ Countries will have the necessary legal and regulatory processes and logistical plans to allow for rapid national or cross-border deployment and receipt of public health and medical personnel during emergencies.*	
			**JEE Technical area 15**<br>National framework for: transferring (sending and receiving) medical countermeasures, and public health and medical personnel from international partners during public health emergencies; and procedures for case management of events due to IHR relevant hazards.	
INT'L COOPERATION ILLICIT TRAFFICKING	***Para 10***<br>*Cooperative action to prevent illicit trafficking*			

CLUSTER	SPECIFIC REQUIREMENTS			IMPLEMENTATION
	**UNSC Res. 1540**	**BTWC**	**IHR (2005)**	
	Cooperative action between states to prevent illicit trafficking in weapons, their means of delivery, and related materials			
SHARING INFORMATION WITH OTHER STATES			***IHR Article 6 (1)***<br>*"Each State arty shall assess events occurring within its territory by using the decision instrument in Annex 2. Each State Party shall notify WHO by the most efficient means of communication available, by way of the National IHR Focal Point and within 24 hours of assessment of public health information of all events which may constitute a public health emergency of international concern within its territory in accordance with the decision instrument as well as any health measure implemented in response to those events..."*<br><br>***JEE Technical area 10: Reporting***<br>Timely and accurate disease reporting according to WHO requirements and	

CLUSTER	SPECIFIC REQUIREMENTS			IMPLEMENTATION
	**UNSC Res. 1540**	**BTWC**	**IHR (2005)**	
			consistent relay of information to FAO and OIE.	
			**IHR Article 6 (1)**<br><br>**JEE Technical area 10**<br>Establishment of a system to report potential public health events of international concern to WHO, and to meet the needs of other official reporting systems, such as OIE-WAHIS.	
SHARING OF EXPIRENCE	***Operative Paragraph 5 of the Security Council Resolution 2325 (2016)*** → *sharing of experiences*	***Article IV***		
	"Encourages also States to prepare on a voluntary basis national implementation action plans mapping out their priorities and plans for implementing the key provisions of resolution 1540 (2004)"	The First Review Conference invited States Parties which have found it necessary to enact specific legislation or take other regulatory measures relevant to this Article to make available the appropriate texts to the United Nations		

CLUSTER	SPECIFIC REQUIREMENTS			IMPLEMENTATION
	UNSC Res. 1540	BTWC	IHR (2005)	
		Centre for Disarmament [now the Office for Disarmament Affairs], for the purposes of consultation...In addition, the Third and Fourth Review Conferences encouraged all States Parties to provide any useful information on the implementation of such measures...<br><br>The Sixth and Seventh Review Conferences also noted that **information** provided to the United Nations by states in accordance with **Resolution 1540** may provide a useful resource for States Parties **in fulfilling their obligations under this Article.**		
PARTICIPATION IN CONVENTIONS,	***Para 1, para 5 and para 8, subpara (a), (b) and (c)***			

CLUSTER	SPECIFIC REQUIREMENTS			IMPLEMENTATION
	UNSC Res. 1540	BTWC	IHR (2005)	
REGIMES, ORGANISATIONS	→ *Adherence to legally binding instruments, membership of organisations, participation in arrangements and statements made.*			
	Promote the universal adoption and full implementation of multilateral non-proliferation treaties (e.g. ratification/accession; participation at meetings; delivering of statements; submission of reports et.)			1. Biological and Toxin Weapons Convention;<br>2. 1925 Geneva Protocol for the Prohibition of the Use of Asphyxiating, Poisonous or Other Gases and of Bacteriological Methods of Warfare;<br>3. 1997 International Convention for the Suppression of Terrorist Bombing;<br>4. 1999 International Convention for the Suppression of the Financing of Terrorism;<br>5. 2005 Protocol to the Convention for the

CLUSTER	SPECIFIC REQUIREMENTS			IMPLEMENTATION
	UNSC Res. 1540	BTWC	IHR (2005)	
				suppression of unlawful acts against the safety of maritime navigation;<br>6. 2005 Protocol to the Convention for the suppression of acts against the safety of fixed platforms located on the continental shelf;<br>7. 2010 Convention on the Suppression of Unlawful Acts Relating to International Civil Aviation;<br>8. Hague Code of Conduct against Ballistic Missile Proliferation
